# Inoculum dose–disease response relationships for the pea root rot pathogen, *Aphanomyces euteiches*, are dependent on soil type and other pathogens

**DOI:** 10.3389/fpls.2023.1115420

**Published:** 2023-05-09

**Authors:** Syama Chatterton, Timothy D. Schwinghamer, Antoine Pagé, Robyne Bowness Davidson, Michael W. Harding, Sabine Banniza

**Affiliations:** ^1^ Lethbridge Research and Development Centre, Agriculture and Agri-Food Canada, Lethbridge, AB, Canada; ^2^ Aquatic and Crop Resource Development, National Research Council Canada, Montreal, QC, Canada; ^3^ Applied Research, Lakeland College, Lacombe, AB, Canada; ^4^ Plant and Bee Health Surveillance, Alberta Ministry of Agriculture and Irrigation, Brooks, AB, Canada; ^5^ Crop Development Centre, University of Saskatchewan, Saskatoon, SK, Canada

**Keywords:** *Aphanomyces* root rot, oospore, inoculum dose, PEA, droplet digital PCR, soil texture

## Abstract

The oomycete pathogen, *Aphanomyces euteiches*, was implicated for the first time in pea and lentil root rot in Saskatchewan and Alberta in 2012 and 2013. Subsequent surveys from 2014 to 2017 revealed that *Aphanomyces* root rot (ARR) was widespread across the Canadian prairies. The absence of effective chemical, biological, and cultural controls and lack of genetic resistance leave only one management option: avoidance. The objectives of this study were to relate oospore levels in autoclaved and non-autoclaved soils to ARR severity across soil types from the vast prairie landscape and to determine the relationship of measured DNA quantity of *A. euteiches* using droplet digital PCR or quantitative PCR to the initial oospore inoculum dose in soils. These objectives support a future end goal of creating a rapid assessment method capable of categorizing root rot risk in field soil samples to aid producers with pulse crop field selection decisions. The ARR severity to oospore dose relationship was statistically significantly affected by the soil type and location from which soils were collected and did not show a linear relationship. For most soil types, ARR did not develop at oospore levels below 100/g soil, but severity rose above this level, confirming a threshold level of 100 oospores/g soil for disease development. For most soil types, ARR severity was significantly higher in non-autoclaved compared to autoclaved treatments, demonstrating the role that other pathogens play in increasing disease severity. There was a significant linear relationship between DNA concentrations measured in soil and oospore inoculum concentration, although the strength of the relationship was better for some soil types, and in some soil types, DNA measurement results underestimated the number of oospores. This research is important for developing a root rot risk assessment system for the Canadian prairies based on soil inoculum quantification, following field validation of soil quantification and relationship to root rot disease severity.

## Introduction

*Aphanomyces euteiches* is the most destructive root rot pathogen of pea in areas with a humid climate (Levenfors et al., 2003). This pathogen is widespread in North America, Europe, Japan, Australia, and New Zealand (Gangneux et al., 2014), but, until 2012, it was not considered a pathogen of concern to pea fields in Alberta and Saskatchewan (Banniza et al., 2013; Chatterton et al., 2015). Intego Solo (a.i., ethaboxam) is the only product registered for early-season suppression of *Aphanomyces* root rot, but it does not reduce disease severity ratings of root rots occurring past the seedling stage (Willsey et al., 2021). Dinitroaniline (e.g., Edge and Treflan) herbicides were effective in Japan and the United States in field trials (Jacobsen and Hopen, 1981), but they are currently not used extensively for disease management. Biological control products were efficacious in field trials conducted in Canada (Xue, 2003), but the suppressive effects can be variable, and there are no commercially available biological control products registered for root rot suppression or management. Incorporation of green manures from Brassicaceae or other soil amendments (e.g., spent lime) suppressed *Aphanomyces* root rot, but the implementation in large-scale field operations is limited (Williams-Woodward et al., 1997; Heyman et al., 2007; Hossain et al., 2012). Therefore, there are currently no efficacious in-crop or preventative treatments available to reduce the impact of *Aphanomyces* root rot. Crop rotation is ineffective in the short term due to the long-term viability of oospores in the soil (Pfender and Hagedorn, 1983), although resistant pulse crops (e.g., faba bean or chickpeas) can be planted instead of susceptible host crops (peas, lentil, alfalfa, and dry bean) (Hughes and Grau, 2007). *Aphanomyces* root rot-resistant pulse crops are not a viable option in all of the growing regions of Saskatchewan and Alberta and may not be attractive alternatives due to market constraints. There are currently no resistant field pea varieties available in North America, although partially resistant germplasm was identified, and new quantitative trait loci were described by Hamon et al. (2013) and McGee et al. (2012). The absence of effective chemical, biological, and cultural control and lack of genetic resistance leave only one management option: growing pulse crops in low-risk fields.

In areas with endemic *A. euteiches* problems, the most recommended practice is disease avoidance based on determining inoculum potential of field soil indexing through greenhouse grow-out tests in field soils (Levenfors et al., 2003; Hughes and Grau, 2007; Sauvage et al., 2007; Gangneux et al., 2014; Harveson et al., 2014). Inoculum potential is an index of potential disease activity of the soil dependent on pathogen infectivity and density and soil factors that can either inhibit or promote infection (Malvick et al., 1994; Moussart et al., 2009). Historically, inoculum potential was determined in a greenhouse bioassay by growing a susceptible pea cultivar in collected field soils under conditions that are conducive to disease development (e.g., seeds treated with metalaxyl and water-saturated conditions; Malvick et al., 1994). A strong positive correlation between disease severities obtained in the greenhouse compared to those observed in the field allows this bioassay to be used as a predictive test. Predictive tests were, however, labor and time intensive, and they often failed to motivate stakeholders, owing to the expense and lack of real-time information. Quantitative molecular techniques like droplet digital PCR or quantitative PCR can be a more efficient method to determine the presence and quantity of *A. euteiches* in soil (Gangneux et al., 2014; Gibert et al., 2021).

Although *A. euteiches* is the most destructive pathogen to pea roots, it often is detected as a complex with other soilborne pathogens (Chatterton et al., 2019). A number of *Fusarium* species were commonly isolated from pea roots in southern Alberta, Canada (Esmaeili Taheri et al., 2017), and *F. avenaceum* and *F. solani* were the most aggressive among tested species (Safarieskandari et al., 2021). Co-inoculation of *A. euteiches* with these two species, and the weakly aggressive *F. redolens*, resulted in statistically significantly higher disease severity ratings compared to single-species inoculation (Willsey et al., 2018). Therefore, the synergistic interactions of *A. euteiches* with other soil pathogens may affect disease severity. In this context, the bioassay may be more predictive, in some cases, than DNA-based analyses of *A. euteiches* alone.

Currently, pulse producers in the Canadian prairies can submit root and soil samples to several commercial laboratories to obtain confirmation on the presence or absence of *A. euteiches*, but no meaningful information on the risk of growing a susceptible crop is provided. A new TaqMan-based multiplex quantitative PCR (qPCR) assay for *A. euteiches*, *Fusarium avenaceum*, and *F. solani* for the purpose of quantifying these pathogens in root tissue (Willsey et al., 2018), a SYBR-green-based qPCR assay (Gangneux et al., 2014), and a ddPCR assay (Gibert et al., 2021) for *A. euteiches* oospores in soil were recently published. There are, however, challenges with detection and quantification of pathogen DNA in soil. First, obtaining representative samples from entire fields is extremely challenging due to field sizes on the Prairies and the irregular distribution of soilborne pathogens. Second, the presence of PCR inhibitors in soil can suppress amplification. Both challenges can lead to false negative results. Adequate sample collection, proper soil preparation, and homogenization can reduce the confounding impact of patchy pathogen distribution in soils. Droplet digital PCR is presumably less sensitive to PCR inhibitors because the inhibitor substances may become sequestered in the individual nano-droplets from DNA molecules (Gibert et al., 2021). As a result, improvements in sample collection, preparation/homogenization, and PCR methodologies can help to ameliorate these challenges.

Field pea is cultivated in a large geographical area across the Canadian prairies, as production spans three major soil zones (black, dark brown, and brown chernozemic soils) owing in part to differences in precipitation, temperature, and native vegetation (Fuller, 2010). Black soils are characterized by high organic matter (5–8.5%) and a low mean annual water deficit of 6.5–13 cm; dark brown by high clay content, moderate organic matter (3.5%–5%), and water deficit (13–19 cm); and the semi-arid brown soil zone with the lowest organic matter (2.5%–3.4%) and highest water deficit (19–38 cm) (Fuller, 2010). Moderate to severe levels of *Aphanomyces* root rot occurs in all of these soil zones (Chatterton et al., 2019). Inoculum potential can be affected by soil type and characteristics (Persson and Olsson, 2000).

With the long-term goal of developing a molecular-based quantification system for measuring *A. euteiches* inoculum potential of prairie soils, the objectives of this study were to 1) relate spiked oospore levels in soils to disease severity for the three common soil zones and types of the Prairies (treatment = three soil zones comprising four soil types); 2) determine whether global soil microbiomes affect the above relationships (treatment = autoclaved or raw (non-autoclaved) soils); and 3) adapt a droplet digital protocol for quantification of *A. euteiches*, *F. avenaceum*, and *F. solani* in soils and use the assay to determine the relationship of measured DNA quantity of *A. euteiches* using ddPCR and qPCR to the starting oospore inoculum dose in soils and determine whether background levels of the two *Fusarium* species affected the disease severity. These objectives support a future end goal of creating a rapid assessment method capable of categorizing root rot risk in field soil samples, aiding producers in pulse crop field selection decisions on the Canadian Prairies.

## Materials and methods

### Soil samples

Soil samples were collected from three soil zones (black, dark brown, and brown) from different fields in Alberta and Saskatchewan in fall of 2015 and 2016. Fields without a history of pulse production were chosen for sampling with the assumption that they would not contain natural inoculum of *A. euteiches*, as the frequency of legumes cropped in a soil is a major indicator of disease risk (Oyarzun et al., 1993). Bulk soil from the top 0–20 cm was collected in large plastic tubs and stored at 4°C. Five days prior to the start of a trial, half of the soil from each location was autoclaved three times at 121°C for 60 min, mixed by shaking the autoclave bag, followed by a 24-h rest period between runs. Soil was autoclaved with the intention of removing any soilborne pathogens and determining the effect of the absence of a global microbiome on disease severity responses. The other half was not autoclaved and served as the raw or non-autoclaved treatment. Soil was then air-dried for 2 days in a drying room so that moisture was roughly equivalent between all soil batches, but the starting soil moisture level was not measured. The soil texture (% sand, silt, and clay) and total percent nitrogen and organic carbon were determined by a commercial soil testing lab (Down to Earth Labs; Lethbridge, AB).

### Preparation of oospores and soil inoculations

Four isolates (Ae1, Ae4, Ae6, and Ae7) of *A. euteiches*, obtained previously from diseased pea roots in Alberta and Saskatchewan (Sivachandra Kumar et al., 2021), were maintained on cornmeal agar (CMA, Sigma-Aldrich, Oakville ON) at room temperature. A mycelia plug of each isolate was transferred to CMA and grown for 3 days, before transfer to homogenized and filtered oatmeal broth in Erlenmeyer flasks (5 plugs/30 ml broth) (Windels, 2000). Each isolate was grown separately with five flasks per isolate. The flasks were then incubated in the dark for 30–45 days. After incubation, the mycelial mats with oospores were homogenized in a Waring blender for 5 min and filtered through four layers of cheesecloth to separate the oospores (Gangneux et al., 2014). The resulting suspension was then centrifuge filtered through 100-µm cell strainers (VWR, Edmonton AB) at 4,000 rpm. The concentration of oospores in the suspension of each isolate was counted using a hemocytometer. The volume of initial oospore suspension of each isolate needed to result in total oospore concentrations of 1,000, 500, 100, 10, and 1 oospores/g soil was calculated, and appropriate amounts to give an equal concentration of each isolate in the mixture were then added to 250 ml of sterile distilled water (SDW). This was then added to 1,250 g of each autoclaved and non-autoclaved soil batch for each target concentration and mixed thoroughly by hand. For the control (0 oospore/g soil), 250 ml of SDW was added to the soils. Square pots (5 cm) were filled with 250 g of soil, with four replicates per treatment, and each pot was placed into a 1-lb plastic bag to catch water run-off and reduce cross-contamination between pots. The experimental layout was as follows: (1) three soil zones (brown, dark brown, and black) collected from one different field per year (2015 and 2016) and per province (Saskatchewan and Alberta) for a total of 12 sources; (2) oospore concentrations, 0, 1, 10, 100, 500, and 1,000 oospores/g soil with an equal amount of each isolate; and (3) autoclaved or non-autoclaved soil. The trial was performed as a randomized complete block design, with all treatment combinations for each year (72 in total per trial) randomized within four trays (24 pots per tray), which were considered to represent one block. Trials were conducted within 2–3 months of collecting the soil and performed twice for each soil source.

### Plant growth and disease rating

Five pea seeds (cv. CDC Meadow) were planted into each pot containing soil prepared as described above. Seeds were surface disinfested for 5 min in 0.5% NaOCl (10% bleach) with a drop of Tween 20 and then washed three times with sterile distilled water (SDW) prior to planting. A preliminary trial was performed to determine whether the different soil types required different watering regimes based on water holding capacity. While some soil types drained faster than others, there did not appear to be any advantage in watering the different soil types with varying volumes of water. As the extra labor and time required did not outweigh small but non-significant differences in disease observed, all experimental pots were watered until run-off every other day. This watering regime kept all soil types sufficiently saturated for disease development. Plants were grown for 5 weeks under standard greenhouse conditions (16:8 h photoperiod, 22°C and 18°C day/night). Roots were washed, and each plant rated for disease on a 0 (no disease)–5 (dead) *Aphanomyces* root rot scale, based on percentage discoloration of the roots, 1 = 1%–25% root discoloration; 2 = 26%–50% root discoloration; 3 = 51%–75% root discoloration; 4 = 76%–100% discoloration; and 5 = dead plant (Papavizas and Ayers, 1974). The disease severity ratings for each pot were converted to a disease severity index (DSI) from 0 to 1 by summing the product of the number of plants in each category by each disease rating category and dividing by the total number of plants rated multiplied by the maximum disease scale. Tests for unequal variance (Levene’s and Bartlett’s) between trials were not significant (JMP 16.0, SAS Institute Inc., Cary, NC), allowing the DSI values from repeated trials for each soil to be pooled for analysis. Although isolations were not performed from all of the roots rated in the experiments due to the overwhelming number of roots generated, random roots from some of the zero oospores soils that showed disease symptoms were plated out after surface disinfestation onto PDA amended with 0.15 g L^−1^ penicillin (Gold Biotechnology, St. Louis, MO, USA) and 0.15 g L^−1^ streptomycin sulfate (Sigma-Aldrich, St. Louis, MO, USA) as described in Esmaeili Taheri et al. (2017). Cultures growing from roots were noted and a presumptive identification made based on colony morphology, but the precise numbers of each colony type were not counted nor were cultures further identified to species.

### DNA extraction and pathogen quantification

Immediately after adding oospores to the soil at the various doses from the samples tested in 2016 only, an aliquot (~50 g) was removed from each treatment batch. This soil was stored at −20°C until processing for extraction. DNA was extracted in duplicate from 250 mg soil samples from each repeated trial (= 4 biological replicates per oospore treatment level), collected from the 50 g retained soil aliquot, using the PowerSoil DNA extraction kit according to manufacturer’s instructions (Qiagen, Toronto, ON). A tetraplex BioRad digital droplet PCR (ddPCR) assay was optimized to quantify three pea root rot pathogens in each DNA extract, using the following targets: the Internal Transcribed Spacer region (ITS) of *Aphanomyces euteiches* (Ae), partial translation elongation factor (TEF) gene of *Fusarium solani* (Fs) and *Fusarium avenaceum* (Fa), and the lipid transfer protein 3 gene from *Triticum aestivum* (TaLTP3) as an internal standard to ensure that amplification had occurred in the event that all of the pathogen targets within a sample were zero (**Table 1**). A total of 10 μl of 10^5^ copies/μl of TaLTP3 gBlock synthetic DNA [Integrated DNA Technologies (IDT), Coralville, IA] was added to each 250 mg soil sample prior to extraction. The optimized parameters included the primer/probe concentration, the template volume (2–8 μl), and the addition of bovine serum albumin (BSA) at high template volumes to eliminate a previously observed “rain” effect (Hughesman et al., 2016). Primer/probes were tested sequentially using different concentrations ranging from 0.25 to 0.75 μM, except for *F. solani*, which was tested up to 1.0 μM, so that two targets could be separated based on amplitude while using the same fluorophore (**Supplementary Table S1**, Biorad, 2016). Higher primer concentrations were assigned to the target that displayed higher fluorescence values during droplet analysis, which helped separate the target droplets with sufficient margin for a clear cut-off value. For Ae and TaLTP3, differing template volumes of 2–8 μl (**Supplementary Table S1**) were added to the reactions to determine if increasing template volume allowed for better detection of the target if present at a low concentration (e.g., low infested field soil). Although detection frequency of low-target copies improved with increased DNA template volume, the “rain” effect increased, which made it difficult to separate out targets (data not shown). BSA was added at low concentrations (**Supplementary Table S1**) to mitigate the “rain” effect from high concentration samples (Biorad technical support personal communication, 2016), lowering the chance of a false positive, but this did not improve detection. Therefore, 2 μl (50 ng total) of template DNA was used for the soil DNA assays. The final optimized 25 µl ddPCR reaction consisted of 12.5 µl ddPCR™ Supermix for Probes no UTP (BioRad, Mississauga, ON), 5.71 µl of primer/probe pool as shown in **Table 1**, 2 µl of sample, and 4.79 µl ddH_2_O. A no template control (NTC) and DNA extracted from oospores of each *A. euteiches* isolate at 10, 100, and 2,500 oospores/ml were included as a positive control. Preliminary testing had indicated that 2,500 oospores/ml was the upper limit of detection, and targets became oversaturated above this level. DNA extracted from 1,000 spores/ml of the two *Fusarium* species was also included as a positive control. The ddPCR reactions were then loaded onto a ddPCR^TM^ 96-well plate, heat sealed using the PX1 plate sealer (BioRad, Mississauga, ON) with pierceable foil heat seal, then loaded onto the QX200 Automated Droplet Generator (AutoDG, BioRad, Mississauga, ON). The AutoDG was loaded as per the specifications of the manufacturer. Briefly, DG32 automated droplet generator cartridges, 2–120 µl pipets for AutoDG system, the sealed ddPCR plate, a cold block with a sample-receiving plate, and automated droplet generation oil for probes were loaded into their respective positions and run. After droplet generation, the sample plate was sealed with foil, then loaded onto the BioRad C1000 touch thermal cycler (BioRad). The ddPCR program was as follows: 98°C for 10 min followed by 40 cycles of 94°C for 30 s and 60°C for 1 min and finally 98 °C for 10 min. The ddPCR plate was then transferred to the QX200 droplet reader (BioRad) for droplet analysis. DNA quantification results were returned as the number of target gene copies per microliter of reaction calculated by the QuantaSoft software (BioRad). The copies per microliter value of the no template control was subtracted from all values of the sample wells before proceeding with analysis.

**Table 1:** Primer and probe sequences and their concentrations used in the tetraplex multiplex assay.

Oligonucleotide Name	Sequence (5`-3`)	Concentration in ddPCR (µM)	Reference
Ae1.2-ITS_Fwd	CCT GCG GAA GGA TCA TTA CC	0.38	Willsey et al. (2018)
Ae1.2-ITS_Rev	AAA ATT ACA TCG GTT CCT TGC G	0.38	
Ae1.2-ITS Probe	56-FAM/TTC TTT ATG/ZEN/AGG CTT GTG CTC TT/3IABkFQ	0.20	
F_Sol_Fwd	GCG CCT TAC TAT CCC ACA TC	1.00	Zitnick-Anderson et al. (2018)
F_Sol_Rev	TTT TGT GAC TCG GGA GAA GC	1.00	
F_Sol_Probe	56-FAM/CCT CCG/ZEN/CGA CAC GCT CT/3IABkFQ	0.50	
FaveSS-Fwd	AAG GCA TGG TGT GA	0.75	designed in house
FaveSS-Rev	TCG CTC TCT GGA AGT TCG	0.75	
Fave-SS-Probe	5-HEX/ACT CCT CGC/ZEN/TAC TAT GTC ACC GTC A/3IABkFQ	0.38	
TaLTP3-178F	GCAGGTGGACTCCAAGCTC	0.38	Foroud (2011)
TaLTP3-320R	GGCACCTGCACGCTATCT	0.38	
TaLTP3 Probe	5-HEX/CTC GAT CAG/ZEN/CAA GGA GTG CT/3IABkFQ	0.20	

Ae, *Aphanomyces euteiches*; F. sol, *Fusarium solani*; F. ave, *Fusarium avenaceum*; TaLTP3, lipid transfer protein 3 gene from *Triticum aestivum*.

To compare the generated ddPCR data for *A. euteiches* to previously published quantitative PCR (qPCR) data (Willsey et al., 2018), analyses were subsequently conducted using a QuantStudio^TM^ Analysis Pro instrument (Applied Biosystems, Mississauga, ON) with the same DNA extracts using the protocol described in Willsey et al., 2018. The Ct values were used to calculate copies per microliter of reaction based on a standard curve using gBlock synthetic DNA [Integrated DNA Technologies (IDT), Coralville, IA] of the target gene sequences from 10 to 10^6^ copies/μl that was included with each qPCR assay run. The generated Ct values were automatically converted to gene copies/microliter by the QuantStudio real-time PCR program (Applied Biosystems) based on the standard curve. For both ddPCR and qPCR, gene copies per microliter were then used to calculate the number of cells per gram of soil based on the assumption that there are 190 ITS copies per *A. euteiches* diploid oospore (Gangneux et al., 2014), which were then transformed using log_10_ + 1 to account for zeroes in the spiked and measured oospore concentration. For estimated concentration of the two *Fusarium* species in soil, the number of TEF1 gene copies per gram soil was log_10_ + 1 transformed prior to statistical analysis.

### Statistical modeling of disease severity index data

Statistical modeling of the DSI data was performed in two steps, both conducted with software suite SAS 9.4 (SAS Institute Inc., Cary, NC). This process was selected to sequentially a) assess the impact that differences in the predictor variables (oospore level, soil zone, soil type, treatment, and year) have on the response variable DSI and b) precisely describe the soil *A. euteiches* oospore level to pea disease severity (DSI) relationships.

The effects of the predictor variables on the response variable DSI were estimated by generalized linear mixed modeling with the GLIMMIX procedure. As the distribution of percentage data is beta-distributed, the beta distribution was specified (DIST = BETA) for the modeling of DSI with the SAS PROC GLIMMIX default logit (log-odds) link function for a beta model. The assumption of variance homogeneity was tested based on the Bayesian information criterion (BIC) goodness of fit estimator. The Gaussian normal distribution of the residuals was not assumed, the models were therefore “generalized.” The fixed effects of oospore level, soil zone, soil type, treatment, year, and the interaction effects on the response variable DSI were evaluated using a series of effect slices. Year was included in the model to account for soils that were collected from the same general location (or closest town) so had the same texture and soil zone profile, but were from a different field that may have had different cropping histories and global microbiome. However, this term also includes the effect of experiment variation, as experiments were performed in the different years in which the soils were collected. The effect of trial was included as an initial term in the analysis, but this term is a covariate of the “texture × type × year” interaction, since different “texture × type × year” combinations were tested in different trials. Effect slices of trial by oospore concentration, treatment, and each texture × type × year combination showed that the measured DSI was different in repeated trials for 10 out of the total of 144 combinations (data not shown). Thus, measurement of DSI was fairly consistent over repeated trials, and subsequently trial was not included in the final model. To visualize the relationship between oospore level and DSI, graphs were produced in SigmaPlot 14.5 using the PROC GLIMMIX estimates of inverse-linked least squares-means and standard errors.

### Comparison of methods for the quantification of *A. euteiches* oospore levels in soil

The *A. euteiches* qPCR (Ct value standard curve to gene copy number) and ddPCR (gene copy number) results were converted to log_10_ (oospores + 1)/g soil so that they could be directly compared to each other and to the starting concentrations of log_10_ (oospores + 1)/g soil applied to the soils. The relationships between oospore levels measured using PCR quantification methods and starting oospore inoculum levels was analyzed using linear regressions, and slopes and intercepts were significant for each regression. The least square means and standard errors for each treatment level was determined using JMP 16.0 using the fit model function, and figures were then produced using Microsoft Excel M365 to visualize the relationship. The effects of PCR type (qPCR or ddPCR), treatment (soil autoclaved or non-autoclaved), and field location, and their interaction on regression parameters [intercept (shifted-*t* distribution) and slope (gamma distribution)] were determined using the GLIMMIX procedure of the statistical software suite SAS 9.4 with output generated from PROC REG estimates of the linear regression intercepts and slopes.

### Quantification of *Fusarium* spp. cell levels in soil

The least square means and standard errors of *F. avenaceum* and *F. solani* log_10_ (TEF1 gene copies + 1)/g soil for each location and treatment level in 2016 were compared using the fit model function in JMP 16.0 and means separated by Tukey’s honestly significant difference (HSD).

## Results

### Soil properties

Although soil was collected from fields according to soil zone, the soil texture analysis revealed that soil zone and soil texture did not always match (**Table 2**). For example, silt loam soils were collected from locations in the black, brown, and dark brown soil zones in Saskatchewan in 2016. Although there was some variation in the percentage of sand and clay between soils from these three locations, they were all comprised of approximately 50% silt. Therefore, for the analysis of the oospore dose–disease response curves, soil type (texture), soil zone, and year were all used as predictor variables to represent each unique location.

**Table 2:** Soil zone, soil type, year, province, closest town, and soil properties for soils used in oospore addition experiments.

Soil Zone	Soil Type	Year	Province	Location	Sand (%)	Silt (%)	Clay (%)	Nitrogen (%)	Organic C (%)
Black	Sandy loam	2015	AB	Lacombe	56.6	27.4	16	0.328	4.035
Black	Silty loam	2016	SK	Melfort	16.5	55.4	28	0.627	6.862
Black	Loam	2015	SK	Rosthern	30.6	47.4	22	0.299	3.525
Black	Loam	2016	AB	Lacombe2	40.5	41.5	18	0.388	4.821
Brown	Loam	2015	SK	Swift Current	38.6	41.4	20	0.142	1.477
Brown	Silty loam	2016	SK	Swift Current2	28.4	51.6	20	0.15	1.459
Brown	Clay loam	2015	AB	Lethbridge	30.6	39.4	30	0.243	2.612
Brown	Clay loam	2016	AB	Rosemary	20.4	43.6	36	0.286	2.775
Dark Brown	Clay loam	2015	AB	Drumheller	32.5	35.5	32	0.468	5.581
Dark Brown	Clay loam	2016	AB	Lethbridge2	28.6	37.4	34	0.304	3.217
Dark Brown	Silty loam	2015	SK	Saskatoon	24.5	55.5	20	0.283	2.915
Dark Brown	Silty loam	2016	SK	Biggar	30.6	51.4	18	0.272	3.531

### Statistical modeling of disease severity index data

**Figure 1:**
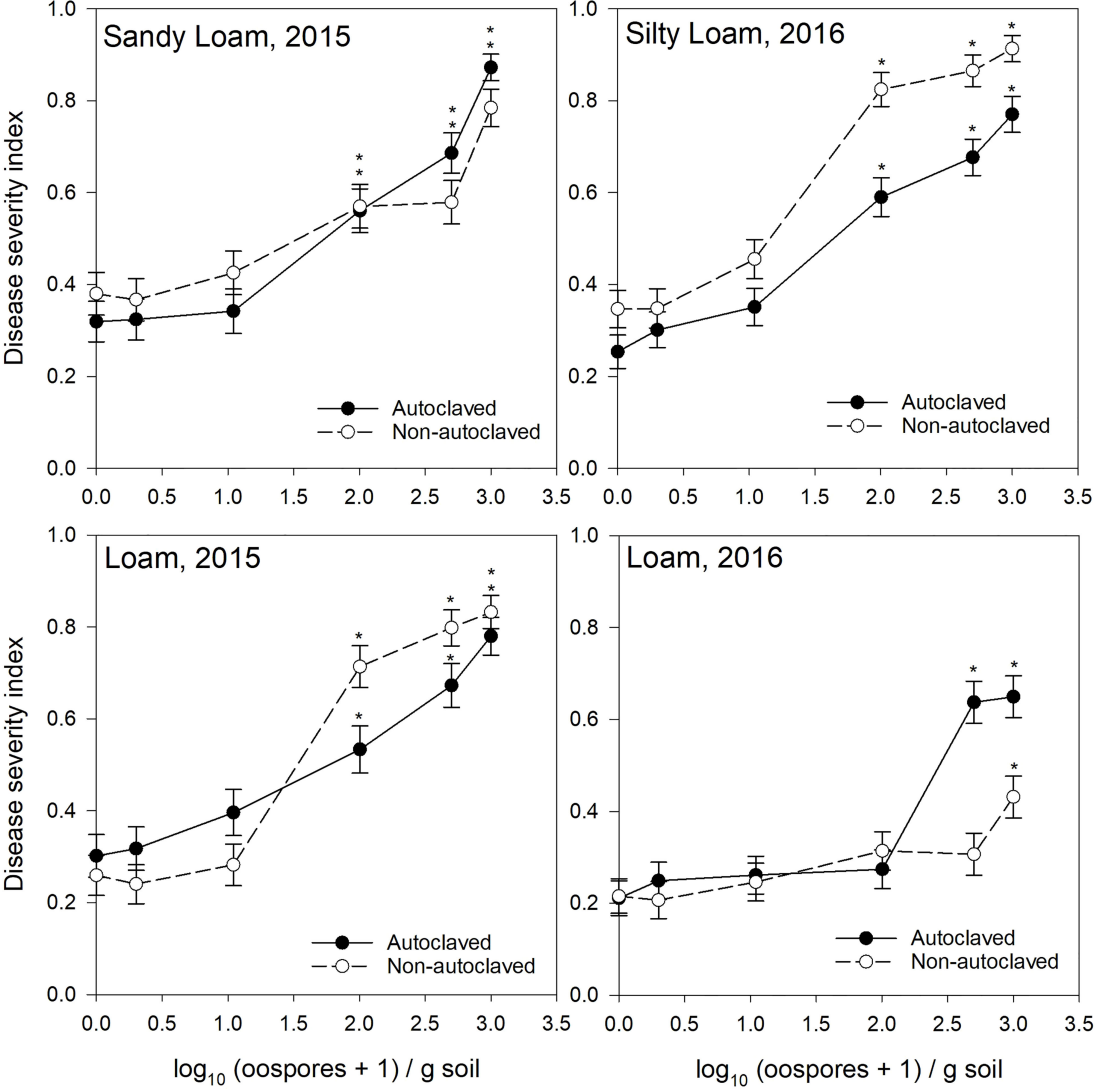
Relationship between spiked oospore concentration (log10 + 1 oospores/g dry soil) and disease severity index of *Aphanomyces* root rot on pea grown in autoclaved or non-autoclaved soils collected from black soil zones. Error bars represent the mean population standard error of the experiment. Asterisks (*) indicate treatment combinations that were significantly different from their respective (autoclaved or non-autoclaved) zero spiked oospore control. ** indicates both autoclaved and non-autoclaved treatments were significantly different from the controls.

**Figure 2:**
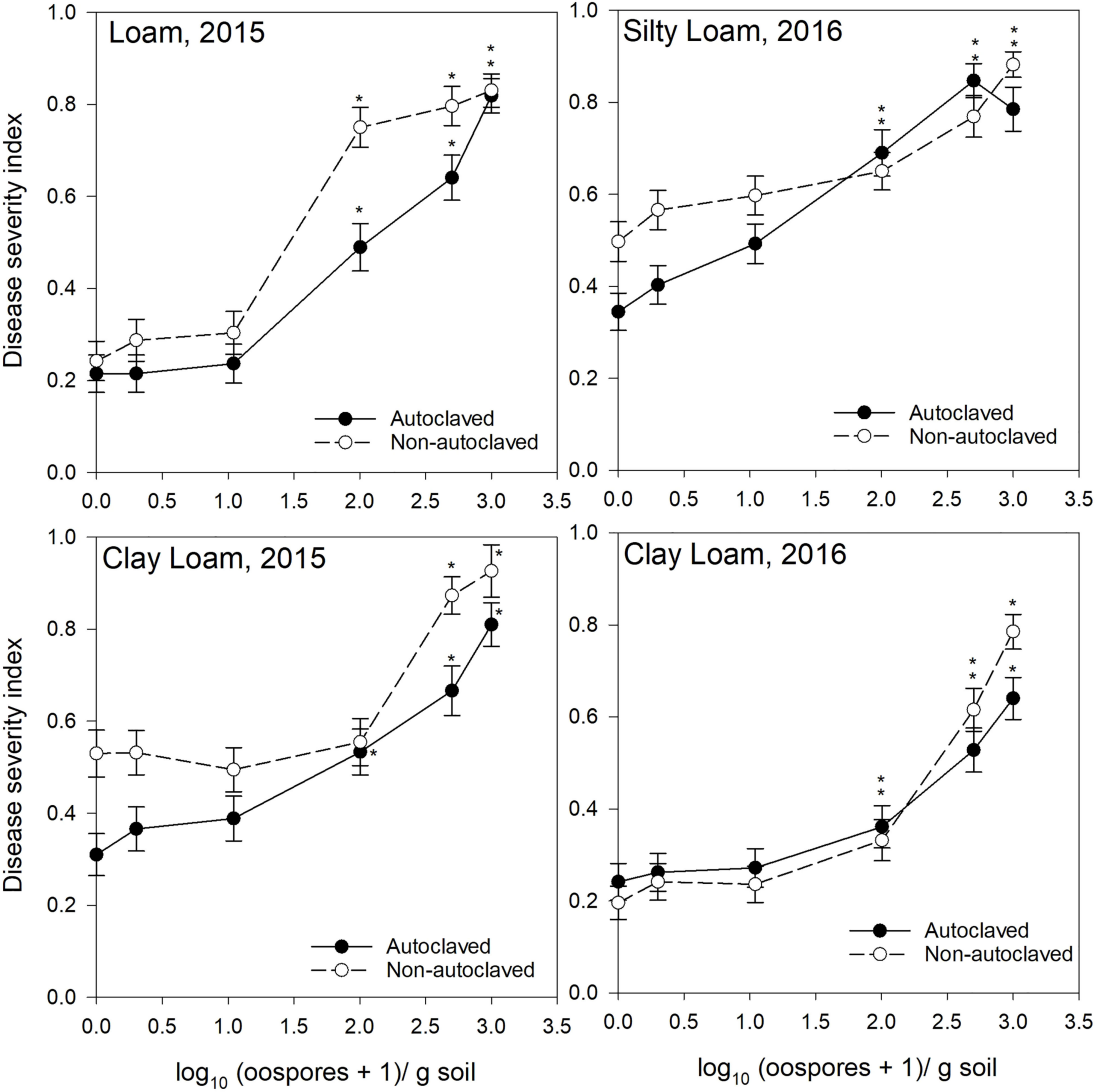
Relationship between spiked oospore concentration (log10 + 1 oospores/g dry soil) and disease severity index of *Aphanomyces* root rot on pea grown in autoclaved or non-autoclaved soils collected from brown soil zones. Error bars represent the mean population standard error of the experiment. Asterisks (*) indicate treatment combinations that were significantly different from their respective (autoclaved or non-autoclaved) zero spiked oospore control. ** indicates both autoclaved and non-autoclaved treatments were significantly different from the controls.

**Figure 3:**
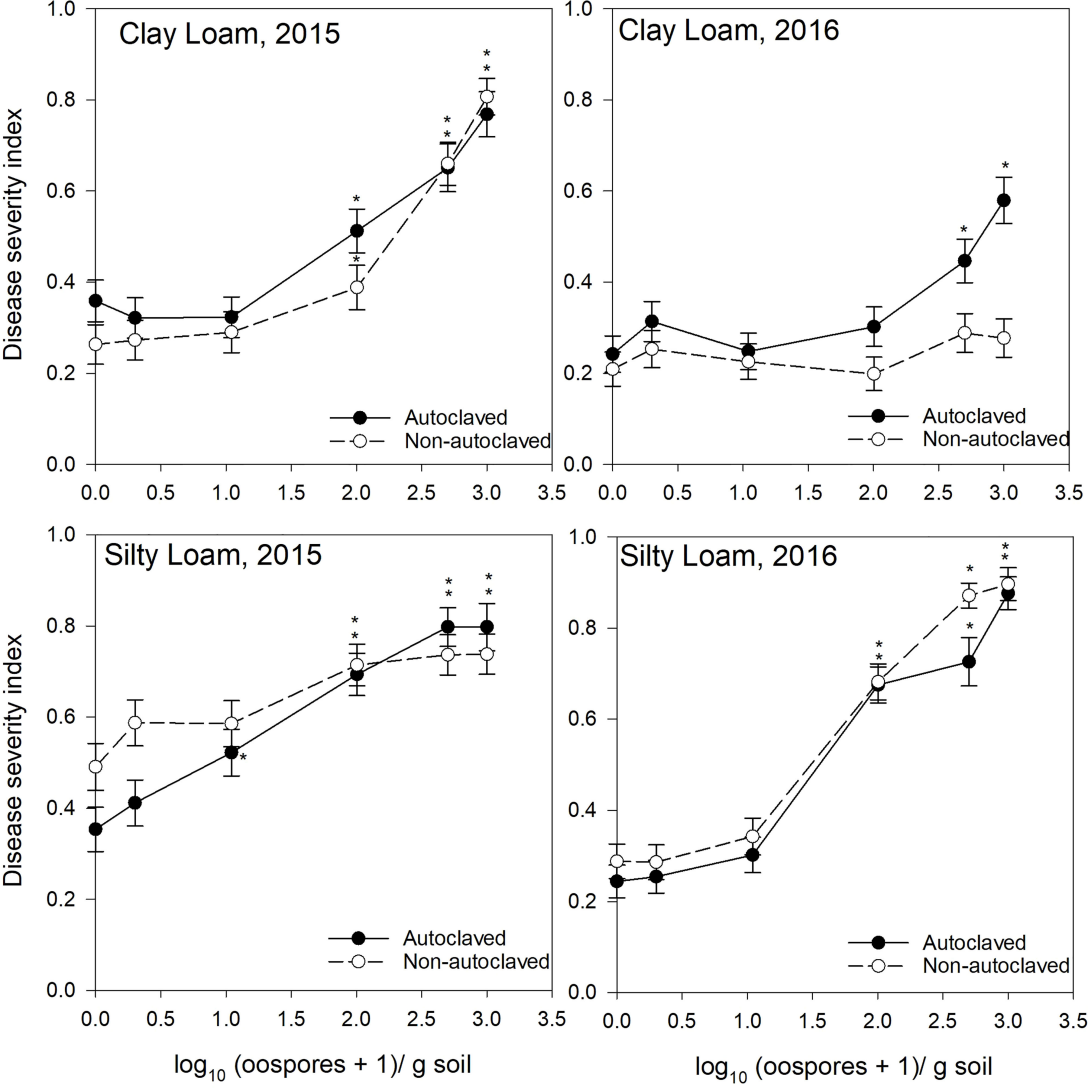
Relationship between spiked oospore concentration (log10 + 1 oospores/g dry soil) and disease severity index of *Aphanomyces* root rot on pea grown in autoclaved or non-autoclaved soils collected from dark brown soil zones. Error bars represent the mean population standard error of the experiment. Asterisks (*) indicate treatment combinations that were significantly different from their respective (autoclaved or non-autoclaved) zero spiked oospore control. ** indicates both autoclaved and non-autoclaved treatments were significantly different from the controls.

The *F*-tests performed to assess the impact of predictor variables on the response variable DSI demonstrated that differences in the value of predictor variables oospore level, soil zone, soil texture, treatment, and year all had significant contributions to the observed variations in DSI (**Table 3**). Several variable interactions were also noted, including the four-way interaction soil zone × soil texture × treatment × year (nested in oospore level). Since this interaction was significant, the GLIMMIX analysis was performed again using location as fixed factor. There was a significant difference between locations, oospore level, and all interaction terms, including location × oospore level × treatment (**Supplementary Table S2**). This test confirmed results of the analysis with the individual terms, but inclusion of the individual terms allowed direct comparisons between factors. Subsequent tests of effect slices indicated that the soil zone, soil texture, and year means of DSI were not equal for various combinations of treatment effects. The tests of effect slices sliced by “soil zone × texture × oospore × treatment” indicated that there were significant differences in DSI between years (2015 and 2016) for the black loam (**Figure 1**), brown clay loam (**Figure 2**), and dark brown silty loam and clay loam soils (**Figure 3**) at several oospore levels in both autoclaved and non-autoclaved soils (**Supplementary Table S3**).

**Table 3:** Type III tests of fixed effects and their interactions included in the nested GLIMMIX analysis of variables that affected the disease severity index of pea grown in soils collected from three soil zones and four soil textures, autoclaved or non-autoclaved (Treatment), and then inoculated with 0,1, 10, 100, 500, or 1,000 oospores/g soil.

Effect	Num DF*	*F* Value	*p*
Year	1	61.08	<.0001
Zone	2	15.90	<.0001
Texture	3	60.60	<.0001
Treatment	1	5.79	0.0164
OosporeLevel	5	257.07	<.0001
Treatment×Year	1	3.14	0.0770
OosporeLevel×Year	5	2.85	0.0148
Zone×Treatment	2	7.57	0.0006
Zone×OosporeLevel	10	1.09	0.3689
Texture×Treatment	3	5.85	0.0006
Texture×OosporeLevel	15	4.33	<.0001
OosporeLev×Treatment	5	0.56	0.7315
Zone×Texture×Treat(Oos)	49	1.97	0.0001
Zone×Texture×Treat×Year(Oos)	17	3.06	<.0001

*Num DF, numerator degrees of freedom. Denominator degrees of freedom was 794.

For the test of effects sliced by “texture × oospore level × treatment × year,” there were significant differences between soil zones within each soil texture. Clay loam soils in 2015 were sampled from brown and dark brown soil zones (**Figures 2**, **3**), and there were significant differences between the DSI responses for non-autoclaved soils only (**Supplementary Table S4**). Silty loam soils were sampled in 2016 from dark brown, brown, and black soil zones (**Figures 1** **–** **3**), and the effect slices indicated that there were significant differences between these soil zones for DSI response at several oospore levels in both autoclaved and non-autoclaved treatments (**Supplementary Table S4**).

To determine the effect of soil texture on the DSI response, effects were sliced by “zone × oospore level × treatment × year.” Loam, sandy loam, and silty loam soils were collected from the black soil zone in 2015 (**Figure 1**), and DSI differed significantly between these three soil textures in non-autoclaved soils at 10, 100, and 500 oospores/g soil only (**Supplementary Table S5**). Loam and clay loam soils were collected in the brown soil zone in 2015 (**Figure 2**), and the DSI differed between these soil textures at all oospore levels from 0 to 100 in both autoclaved and non-autoclaved treatments, except the autoclaved 0 treatment. Clay loam and silty loam soils were sampled from the brown soil zone in 2016 (**Figure 2**), and DSI differed between these two soil types at all oospore and treatment levels, except the autoclaved 0 level. Clay loam and silty loam soils were sampled from the dark brown soil zone in 2015 (**Figure 3**), and there were significant differences between these soil types at several oospore levels in the autoclaved and non-autoclaved treatments.

For the effect of treatment, there were significant differences between autoclaved and non-autoclaved soils for the following combinations: black loam soil in 2015 at 100 and 500 oospores/g soil; black silty loam in 2016 at 100, 500, and 1000 oospores/g soil; brown clay loam in 2015 at 0, 1, and 500 oospores/g soil; and brown clay loam in 2016 at 1,000 oospores/g soil; brown loam in 2015 at 100 and 500 oospores/g soil; brown silty loam in 2016 at 0 and 1 oospores/g soil; dark brown silty loam in 2015 at 1 oospore/g soil; and dark brown silty loam in 2016 at 500 oospores/g soil (**Supplementary Table S6**). The test of effects sliced by “zone × texture × treatment × year” indicated that there were significant differences between oospore levels for all combinations, and the differences were explored further using simple effect comparison of the means using Scheffe’s multiple grouping method.

Low to moderate levels (0.2–0.4 DSI) of disease were observed in the control autoclaved and non-autoclaved soils for all of the “soil zones × texture” combinations (**Figures 1** **–** **4**). For all “soil zone × textures,” there was no statistical difference between 0, 1, and 10 oospore levels, except at 10 oospores/g soil in the dark brown silty loam 2015 soil (**Figure 3**). The DSI at 100 oospores/g soil was significantly higher than the DSI at 0, 1, or 10 oospores/g soil at the following locations and treatments: black silty loam 2016, black loam 2015, dark brown silty loam 2016, and brown loam 2015. For all other locations, except black loam 2016 and dark brown clay loam 2016, the DSI at 100 oospores/g soil was between that at 10 and 500 oospores/g soil and was above 0.5 DSI. In the dark brown clay loam 2016 (Lethbridge2) soil, there was no difference between DSI at any of the oospore levels in the non-autoclaved treatment (**Figure 3**). For black loam 2016 (Lacombe2), only the DSI at 1,000 oospores/g in the non-autoclaved soil was significantly higher from all of the other oospore levels (**Figure 1**). For almost all “soil zone × texture” datasets, the maximum DSI ranged from 0.8 to 0.97 at the highest oospore level of 1,000 oospores/g soil (**Figures 1** **–** **3**). The exceptions were dark brown clay loam 2016 (Lethbridge2) and black loam 2016 (Lacombe2), where maximum disease severity was 0.26 and 0.44, respectively, in the non-autoclaved soil, and 0.58 and 0.66, respectively, in the autoclaved soil, at 1,000 oospores/g soil.

**Figure 4:**
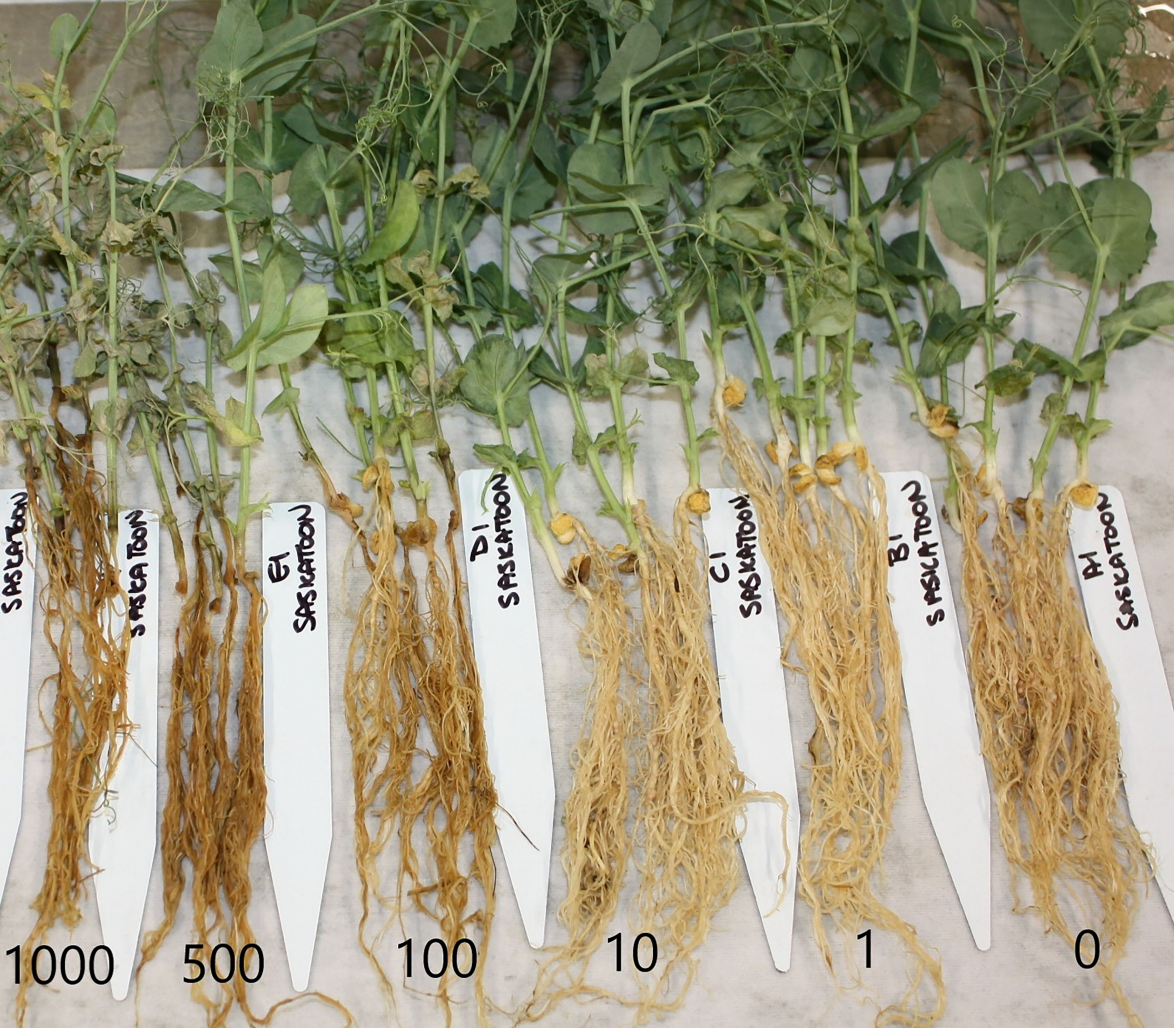
Disease symptoms on pea plants grown in non-autoclaved soil from Saskatoon (dark brown silt loam) in 2015 inoculated with 0 (right) – 1,000 (left) oospores/g soil.

### DNA quantification of *A. euteiches* levels in soil

**Figure 5:**
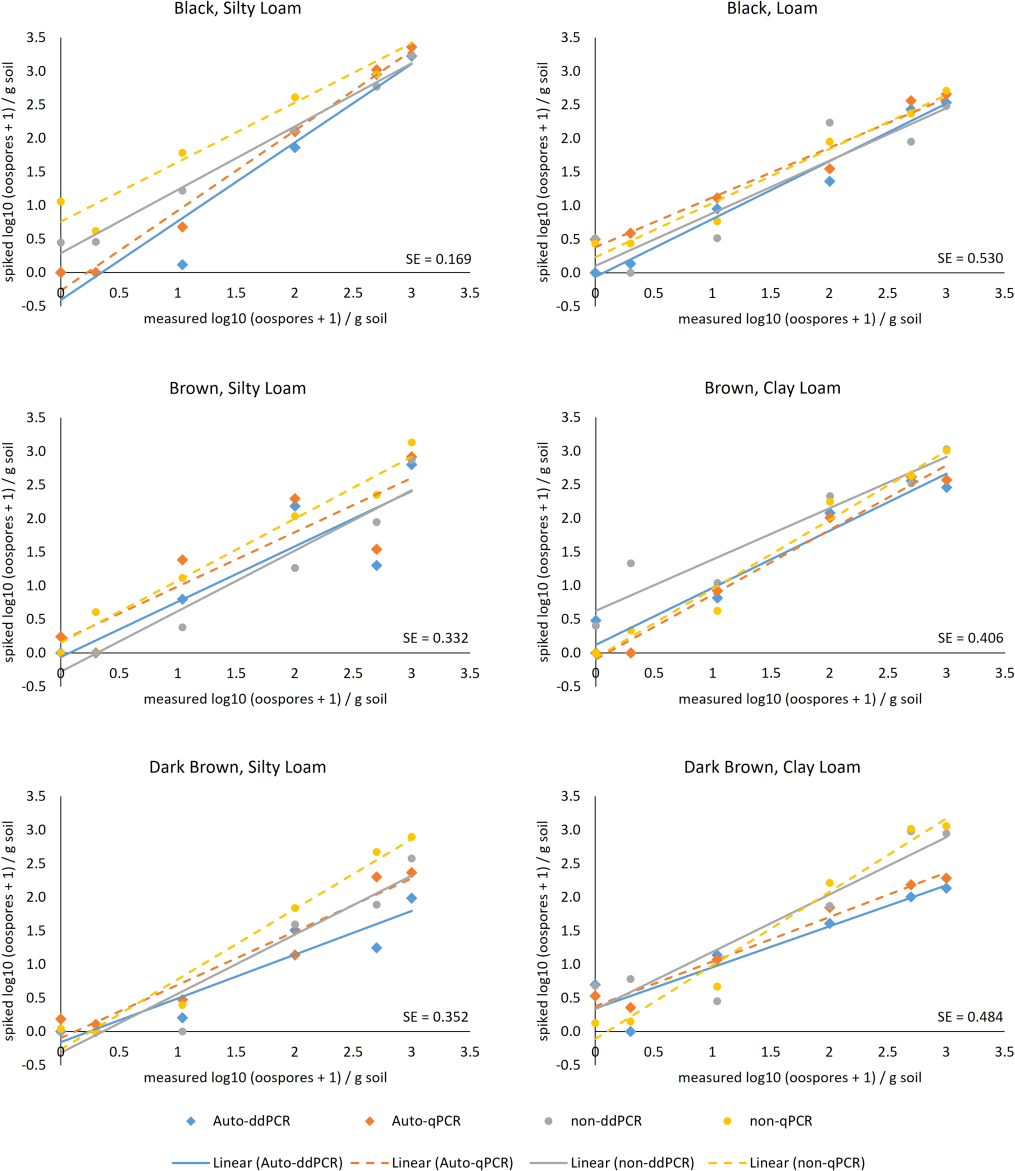
Relationship between number of oospores/g soil calculated from ddPCR (solid line) and qPCR (dashed line) analysis of DNA extracted from soils that were autoclaved (diamonds) or non-autoclaved (circles) from six locations (= unique soil zone and texture) with 0, 1, 10, 100, 500, and 1,000 oospores added. Oospore levels were calculated from ITS copies/microliter for ddPCR and ITS copies standard curve (*via* Ct values) for qPCR, based on the assumption of 190 ITS copies per oospore and a DNA extraction from 250 mg soil. Standard error bars are not shown, but the mean population standard error (SE) for each location is given in the lower right-hand corner of the graph.

The type III tests of fixed effects [field location, PCR type (ddPCR and qPCR), and treatment (autoclaved or non-autoclaved soils)] of the slopes determined from linear regressions between measured oospores and added oospores (**Figure 5**) showed that all factors, including location (soil zone × texture), and their interactions were significant (**Table 4**). There was significant difference in the slopes of the regression lines calculated for qPCR and ddPCR for dark brown silty loam, dark brown clay loam, and brown clay loam, with the slope of the qPCR line higher than those of the ddPCR lines (**Figure 5**, **Supplementary Figure S1A**, **Supplementary Table S8**). The slopes for the dark brown clay loam (Lethbridge2) autoclaved ddPCR and qPCR lines were the lowest at 0.61 and 0.66, respectively, indicating significant underestimation of oospore levels in the soil compared to the actual amounts added (**Supplementary Figure S1A**, **Supplementary Table S8**). The slopes for the black silty loam (Melfort) autoclaved qPCR and ddPCR lines were the highest at 1.19 and 1.17, respectively, and R^2^ values were 0.94 and 0.98 (**Supplementary Figure S1A**, **Supplementary Table S8**). For treatment × field location interactions, there were significant differences in the slopes of the lines for autoclaved and non-autoclaved soils from dark brown silty loam, dark brown clay loam, black silty loam, and brown silty loam locations (**Figure 5**, **Supplementary Figure S1A**). For dark brown silty loam, dark brown clay loam, and brown silty loam, the slopes for the autoclaved soil lines were lower than the non-autoclaved lines, but the reverse was true for black silty loam.

**Table 4:** Type III tests of the fixed effects of field location (= unique soil zone × texture), PCR type (qPCR or ddPCR), and treatment (autoclaved or non-autoclaved) on the regression parameters for the regression analysis of log10 (oospores +1)/g soil added to the soils versus the calculated concentration of log10 (oospores +1)/g soil measured in soil using qPCR or ddPCR.

Effect	Numerator DF*	*F* Ratio	*p*
Slope (*b_1_*)			
Location	5	66.40	0.0001
PCR type	1	58.48	0.0006
Treatment	1	73.38	0.0004
Location×PCR type	5	22.30	0.0020
Location×Treatment	5	89.36	<.0001
Treatment×PCR type	1	13.43	0.0145
Intercept (*b_0_*)			
Location	5	14.59	0.0053
PCR type	1	0.69	0.4428
Treatment	1	2.36	0.1854
Location×PCR type	5	7.58	0.0221
Location×Treatment	5	4.31	0.0673
Treatment×PCR type	1	3.10	0.1385

*Denominator degree of freedom = 5.

The type III tests of fixed effects [field location, PCR type (ddPCR and qPCR), and treatment (autoclaved or non-autoclaved soils)] of the intercepts calculated from linear regressions between measured oospores and added oospores (**Figure 5**) revealed that only location and the interaction between location and PCR type were significant (**Table 4**). The intercepts for black loam, brown clay loam, and brown silty loam were significantly different between qPCR and ddPCR (**Supplementary Figure S1B**). Although there was a large numerical difference between the intercepts for the autoclaved and non-autoclaved black silty loam soil, this difference was not significant due to the large upper and lower confidence limits (**Supplementary Figure S1B**). The intercepts ranged from as low as −0.41 (ddPCR autoclaved, black silty loam) to as high as 0.76 (qPCR non-autoclaved, black silty loam) (**Supplementary Figure S1B**, **Supplementary Table S8**).

### *Fusarium* levels in soil

**Figure 6:**
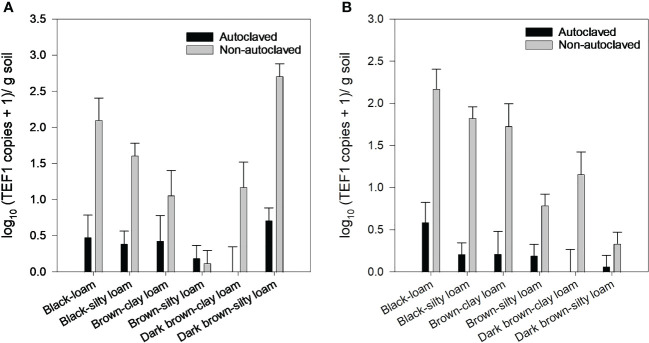
*Fusarium avenaceum* **(A)** and *Fusarium solani* **(B)** concentration [log_10_ (TEF1 gene copies + 1)/g soil] in soils from six locations (=unique soil zone and texture) that were autoclaved or non-autoclaved from 2016 soil collections. Error bars represent the mean population standard error of the experiment.

Levels of *F. avenaceum* and *F. solani* were quantified in soils collected in 2016 using ddPCR. *F. avenaceum* was present in soils from all locations, but levels were very low in brown silty loam (**Figure 6**). *Fusarium solani* was also present in all soils, but levels were lower in brown silty loam and dark brown silty loam than the other soils (**Figure 6**). Autoclaving soils significantly reduced the levels of *F. avenaceum* and *F. solani* compared to the non-autoclaved soils but did not completely eliminate their DNA from soils. Although we did not perform isolations from all roots in all of the trials, periodic plating of random root samples from the zero oospore treatments yielded various *Fusarium* species, primarily presumptive *F. avenaceum*, *F. solani*, and *F. redolens* based on colony morphology and common saprophytes like *Rhizopus* and *Penicillium* spp. (data not shown).

## Discussion

The primary objective of this study was to relate oospore levels of *A. euteiches* in soil to disease severity for the common soil zones of the Prairies. Care was taken to select a balanced number of fields in each soil zone (black, dark brown, and brown) in each province and year for subsequent testing of the inoculum dose–disease response relationship. However, because there were different soil textures across soil zones, this resulted in an unbalanced design when accounting for soil texture × zone interactions. Thus, for statistical analysis, generalized linear mixed modeling with nested factors was used to account for the unbalanced design. This analysis, along with the graphical representation of disease severity levels, clearly showed that there was a differential disease severity outcome to oospore concentrations for each of the different soil textures and zone combinations. Therefore, although the intention of this research was to develop a generalized disease severity–oospore dose model, the nature of the interaction of disease development with the large array of soil zones and textures within the Canadian prairies renders this relationship more complex.

Soil zones are defined by their biogeographic properties that include differences in annual precipitation, temperature, organic matter, and native vegetation (Fuller, 2010), all of which will affect the soil microbiome and ecology. Soil texture, on the other hand, refers to the percent composition of silt, clay, and sand. The percentage of these components affect water holding capacity and drainage, and the physical nature of oospore interactions with soil particles. The combination of both soil zone and texture defines the soil’s physicochemical properties, which are known to affect *Aphanomyces* root rot development (Persson and Olsson, 2000). Thus, it was not surprising that both soil zone, texture, and their interaction resulted in differential DSI responses to oospore concentrations in soil. For example, some glacial clay soils (35%–40% clay content) were more conducive to *Aphanomyces* root rot of pea than till clay soils due to their different source material and thus different physicochemical properties (Persson and Olsson, 2000). Generally, high clay soils are more compact with low water permeability, which favors root infection by zoospore-producing pathogens (Persson and Olsson, 2000).

Year was also included in the model, as soils were collected in two different years, and the experiments with each soil set were also performed in two different years. This term was thus included in the model because soil collection year could have affected biological properties (e.g., the global microbiome) of the soil. The year 2015 was warmer and drier than average in Alberta and Saskatchewan, while 2016 was wetter and cooler than average resulting in higher root rot prevalence and incidence in 2016 (Chatterton et al., 2019). Although soils were dried prior to spiking with oospores, differences in weather and local edaphic condition soils experienced prior to collection could have affected microbial community composition (Bainard et al., 2016); for example, frequency of pea root rot pathogens was affected by year (Esmaeili Taheri et al. 2017). In addition to the influence of weather on soil microbial communities, the effect of year on experimental variance cannot be fully discounted. Soils collected in 2015 were performed as one experimental batch with two repeated trials, and those collected in 2016 were performed as a separate experimental batch. Therefore, the significance of year in the model could also be due to the variation between experiments. The precise differences in specific soil properties as a combination of soil texture, soil zone, and year (weather and edaphic factors) that account for the differential disease–response relationship observed in this study should be explored further but were beyond the scope of this project.

Even within similar soil zone × texture groups, there were dissimilar responses for the Lethbridge2 (dark brown and clay loam) and Lacombe2 (black and loam) soils from the other locations within their respective soil groupings. Disease severity in these two soils remained low at almost all oospore levels, including 1,000 oospores/g soil, suggesting a suppressive soil effect. Oospore inoculations of the autoclaved soils resulted in some, but not complete, restoration of higher disease levels, suggesting that the suppressive effect is both biotic and abiotic. Both of these fields had a history of compost application. Although compost has been linked to building suppressive soils (Hadar and Papadopoulou, 2012), further investigations of these soils is required to confirm the suppressive effect and elucidate mechanisms.

The other major factor affecting the disease severity–dose response relationship was whether the soil had been autoclaved prior to inoculations. Although autoclaving can alter soil properties (Berns et al., 2008), the primary purpose of autoclaving the soil was to eliminate any other native pathogens in the soil and to also determine the effect of the global soil microbiome by comparing the response between autoclaved and non-autoclaved soils. The response to autoclaving also varied by soil texture and zone. For some soil zones × texture (clay loam–dark brown and brown, and silty loam–dark brown), there was no difference in disease severity at the various oospore levels between autoclaved and non-autoclaved soils, whereas for other locations, disease severity at each oospore concentration was generally higher in non-autoclaved treatments than autoclaved treatments. This could indicate that other organisms within the soil contribute to enhancing disease, although the confounding effects of autoclaving on changing the soil parameters cannot be fully discounted. However, assessment of two *Fusarium* species that are commonly associated with the pea root rot complex (Esmaeili Taheri et al. 2017, Chatterton et al., 2019) showed that *F. avenaceum* and *F. solani* were present at higher levels in all of the non-autoclaved soils, although concentration varied between soils. Although we did not perform isolations from all roots from all of the locations, presumptive *F. avenaceum*, *F. solani*, and *F. redolens* isolates, based on colony morphology, were observed on root pieces in culture, as were common saprophytes like *Rhizopus* and *Penicillium* spp. (data not shown). In greenhouse trials, co-inoculation of *A. euteiches* with *F. avenaceum* and/or *F. solani* resulted in significantly higher disease severity levels than any of the pathogens occurring singly (Willsey et al., 2018).

One of the challenges with interpreting the results from this study is that root browning was often observed in the non-inoculated (zero oospores/g soil) treatments for all soils. These roots were often scored with a disease rating of 1 (<25% of roots browned), but it was difficult to determine if it was due to pathogen infection or root staining from the soils. This was observed even in autoclaved soils, and for the most part, disease severity did not differ between the autoclaved and non-autoclaved soils without oospore treatments. The exception was in silty loam soils where the non-autoclaved soils had a higher disease severity than the autoclaved soils without any addition of oospores. The silty loam soils were all collected in 2016, and all of these soils had *F. avenaceum* and/or *F. solani* at various levels. Soils were collected from fields that did not have any prior history of *A. euteiches*, with the assumption that they would be free from *A. euteiches*, as soils with a cropping history of pulses are at higher risk of *A. euteiches* infestation (Pfender and Hagedorn, 1983). It is possible that some soils may have had low levels of *A. euteiches*, since research in Saskatchewan showed that soils from native pastures can contain low levels of *A. euteiches* (Karppinen et al., 2020). This seems particularly likely for the black silty loam soil that showed *A. euteiches* in the non-autoclaved, non-inoculated treatment in both the qPCR and ddPCR results. Other pathogens such as *Pythium* spp., *Rhizoctonia solani*, or other *Fusarium* spp. may also have been present in the soil or on the seed and confounded disease severity ratings.

Visual representation of the relationship between oospore dose and disease severity clearly showed that the relationship was not log-linear. Linear regression using the whole data set was attempted but resulted in a low *R* ^2^ value (data not shown), likely due to the differential responses for DSI between soil zones × textures. Previous research with soils from France and Sweden showed a log-linear relationship between oospore dose and disease severity (Persson et al., 1999; Sauvage et al., 2007; Gangneux et al., 2014). In our study, either disease did not develop, or severity was not significantly different from zero oospores, when oospore levels were below 100 oospores/g soil for all soil zone by textures. Similar to these previous studies, we did observe that disease reached a maximum level (i.e., DSI = 1) at 1,000 oospores/g soil for several soil types. Previous studies used a larger range of oospore concentrations, but fewer soil sources, and soils were only inoculated with one *A. euteiches* isolates (Sauvage et al., 2007; Gangneux et al., 2014). In our study, we inoculated soils with a mixture of four *A. euteiches* isolates and used a smaller range of oospore concentrations because of the large number of soils that were being evaluated. In the range of 10 of 1,000 oospores, which is comparable to these other studies, a linear relationship was apparent for some soils. There can be a significant variation in aggressiveness among *A. euteiches* isolates (Sivachandra Kumar et al., 2021), so it is possible that using a mixture of isolates contributed to the non-linear relationships observed. Of the four isolates that were used in this study, three (Ae4, Ae6, and Ae7) were highly aggressive towards CDC Meadow, and one (Ae1) was moderately aggressive, while two isolates (Ae6 and Ae7) also caused moderate disease severity on the partial resistant line PI660736 (Sivachandra Kumar et al., 2021). Furthermore, while care was taken to ensure that there was an equal concentration of oospores from each isolate in the inoculation mix, our personal observations repeatedly working with these isolates is that some consistently produce more oospores and zoospores than others (e.g., Ae1 produces more zoospores but fewer oospores than Ae6). These intrinsic properties of the different isolates could also affect resulting disease severity.

Finally, we also compared the use of qPCR and ddPCR to quantify *A. euteiches* DNA in the initial soil dilution series to determine whether these tools can be used to accurately measure oospore concentrations in different soils. In order to compare qPCR and ddPCR, the returned Ct values (qPCR) and ITS copy number per microliter (ddPCR) were converted to an estimate of oospore numbers per gram of soil. Although the ITS copy number is variable among isolates (Gangneux et al., 2014), we used an average of 190 ITS copies per diploid cell for ease of calculations and because it is close to the mean of 95 ± 22 copies per cell calculated for 40 *A. euteiches* isolates (Gangneux et al., 2014). More precise measurement of actual copy number per cell was described by Gibert et al. (2021) by also using a single-copy *A. euteiches* gene target (Sauvage et al., 2007), but this method does not work well for soil due to the low sensitivity of quantifying a single-gene target sequence. For the purposes of estimating *A. euteiches* inoculum levels in soil and developing a test that can easily be implemented by commercial labs, our results show that using an average of 190 ITS copies per cell for calculating oospores per gram soil works well, since for most soils, there was a significant correlation between initial oospore concentration and calculated oospore concentration, with slopes close to 1. As expected, quantification below 10 oospores/g soil was not accurate, as this quantity is reaching the theoretical limit of detection from 250 mg of soil (Willsey et al., 2018) and resulted in the intercepts for several soils falling below or above zero. Digital droplet PCR has the potential to be more sensitive for quantifying DNA of a relatively rare target in soil than qPCR (Gibert et al., 2021). However, in the side-by-side comparison of qPCR and ddPCR amplification of inoculated oospores, for several soil types, the slope of the line for the ddPCR assays was significantly lower than for the qPCR assays. In most cases, this resulted in an underestimation of oospores per gram of soil compared to the actual amount for the ddPCR assays. Gibert et al. (2021) used 200 ng of soil matrix DNA per PCR mixture in order to obtain increased sensitivity. In our study, increasing the amount of soil DNA resulted in a greater rain effect, which inhibited differentiation of the four targets in the multiplex assay, and thus, only 50 ng of total soil DNA was used per reaction. On the other hand, the qPCR assay was performed as a singleplex for *A. euteiches* only, and thus, it is possible that some sensitivity was lost in the multiplex ddPCR assay. However, a multiplex assay that can target multiple species within the root rot complex would be beneficial for reducing per sample assay costs and for more precise risk prediction, given that multiple species interact together to increase disease severity. Therefore, further research into enhancing the sensitivity of a multiplex ddPCR assay would be beneficial.

Both qPCR and ddPCR assays were affected by soil texture × soil zone and autoclave treatment. For ddPCR, the black silt loam soil had the highest *R* ^2^ and slope closest to 1, while the dark brown clay loam soil had the lowest *R* ^2^ and slope. For qPCR, dark brown silt loam, black silt loam, and brown clay loam soils had the highest *R* ^2^ values and slopes closest to 1, while the black loam soil had the lowest *R* ^2^ and slope. Although organic matter, humic acid, and clay content can all affect DNA quantification results from soils (Frostegård et al., 1999; Almquist et al., 2016; Gibert et al., 2021), there did not appear to be any clear trends on the effects of these factors with the soils we tested. The two black soils had the highest organic matter, but the black silt loam soil performed the best for ddPCR and qPCR, while the black loam soil performed the worst. Similarly, the two clay loam soils had contrasting performance for both qPCR and ddPCR assays. The finding that soil type influences oospore quantification has been described for related species, *Aphanomyces cochlioides* where oospore detection limits were higher in high clay soils (Almquist et al., 2016) and *Phytophthora medicaginis* where quantification was lower in sand than in soil (Bithell et al., 2021). In terms of the effect of autoclaving, dark brown silt loam, black silt loam, and dark brown clay loam soils had significantly different slopes between autoclaved and non-autoclaved treatments, even though oospores were added after autoclaving. Autoclaving can affect soil properties, causing, for example, a decrease in aggregation, a corresponding increase in the clay fraction, and more dissolved organic matter (Berns et al., 2008). Changes in these properties could have affected performance of the ddPCR and qPCR reactions, although it is not clear why only some soils were affected. Autoclaved soils could also have a different microbiome if the native soil microbiome was rapidly replaced by fast colonizers. As discussed above, the disease severity and oospore dose relationship may also have been affected by changes in soil properties due to autoclaving, but other soil sterilization procedures may also result in changes to soil properties (Berns et al., 2008), and other sterilization equipment are not as readily available as an autoclave. It is possible that this differential effect was due to the different spiking events and random variation in oospore distribution when collecting 2 × 250 mg samples from each spiking event. Taken together, the results for the effects of soil texture and zone and autoclaving suggest that soil properties may affect the performance of both qPCR and ddPCR, although the exact nature requires further research. This is being tested with more replicates on a larger number of soil samples to determine if random variation in sampling is the biggest factor.

This research demonstrated that developing a model for predicting severity of *Aphanomyces* root rot based on DNA quantification of soils will not be an easy task for the large geographical area under which pea is cultivated in the Canadian prairies. The vast area encompasses several biogeographical zones and soil types. Our research clearly showed that the relationship between disease severity and oospore concentration was different based on soil zone and texture. In addition, the presence of other pathogens and potentially, beneficial organisms, in the soil further complicates this relationship. Furthermore, using DNA quantification tools to estimate initial oospore concentration in the soil was also affected by soil properties. Nonetheless, this research is the first step towards understanding inoculum thresholds that are required for disease progression in Canadian prairies soils and defining the relationship between pathogen inoculum and disease severity. Further research to better understand the factors that affect DNA quantification accuracy and sensitivity is currently underway by testing a much larger set of soils from across the Canadian prairies.

## Data availability statement

The raw data supporting the conclusions of this article will be made available by the authors, without undue reservation.

## Author contributions

SC performed the research and wrote the manuscript, TS analyzed the data, AP assisted with manuscript writing, RD, MH and SB provided soil samples and manuscript review and editing. All authors contributed to the article and approved the submitted version.
