# Supplementary material for: Inoculum dose–disease response relationships for the pea root rot pathogen, Aphanomyces euteiches, are dependent on soil type and other pathogens
Source: Front Plant Sci. 2023 May 9;14:1115420. doi: 10.3389/fpls.2023.1115420 (PMC10205554; doi:10.3389/fpls.2023.1115420)

| Supplemental Table 1. Optimization parameters for the ddPCR quadplex assay | | | | | | | | | | | | | | | | |  |  |  |  |
| --- | --- | --- | --- | --- | --- | --- | --- | --- | --- | --- | --- | --- | --- | --- | --- | --- | --- | --- | --- | --- |
| **Target** | | **Primer Concentration (µM)** | | | | | **Probe Concentration (µM)** | | | | | **Template (µL)** | | | | **BSA (µg/mL)** | | | | |
| Ae | | 0.25 | 0.380 | 0.50 | 0.75 |  | 0.125 | 0.200 | 0.25 | 0.375 |  | 2 | 4 | 6 | 8 | 0 | 0.02 | | 0.05 | 0.1 |
| Fs | | 0.25 | 0.375 | 0.50 | 0.75 | 1.00 | 0.125 | 0.188 | 0.25 | 0.375 | 0.50 |  |  |  |  | 0 | 0.02 | | 0.05 | 0.1 |
| Fa | | 0.25 | 0.375 | 0.50 | 0.75 |  | 0.125 | 0.188 | 0.25 | 0.375 |  |  |  |  |  | 0 | 0.02 | | 0.05 | 0.1 |
| TaLTP3 | | 0.25 | 0.380 | 0.50 | 0.75 |  | 0.125 | 0.200 | 0.25 | 0.375 |  | 2 | 4 | 6 | 8 | 0 | 0.02 | | 0.05 | 0.1 |

Table S2. Type III tests of fixed effects when location is included in the GLIMMIX analysis of DSI.

| **Type III Tests of Fixed Effects** | | | | |
| --- | --- | --- | --- | --- |
| **Effect** | **Num DF** | **Den DF** | **F Value** | **Pr > F** |
| **OosporeLevel** | 5 | 1032 | 357.40 | <.0001 |
| **Treatment** | 1 | 1032 | 1.84 | 0.1756 |
| **OosporeLev*Treatment** | 5 | 1032 | 5.30 | <.0001 |
| **Location** | 11 | 1032 | 86.09 | <.0001 |
| **OosporeLevel*Location** | 55 | 1032 | 9.93 | <.0001 |
| **Treatment*Location** | 11 | 1032 | 19.28 | <.0001 |
| **Oospore*Treatme*Location** | 55 | 1032 | 5.23 | <.0001 |

Table S3. Tests of effect slices sliced by ‘soil zone x texture x oospore x treatment’ to determine effect of year on DSI.

| **Tests of Effect Slices for Zon*Typ*Tre*Yea(Oos) Sliced By Zone*Type*Trea(Oosp)** | | | | | | | |
| --- | --- | --- | --- | --- | --- | --- | --- |
| **Zone** | **Type** | **Treatment** | **OosporeLevel** | **Num DF** | **Den DF** | **F Value** | **Pr > F** |
| black | loam | Auto | 0 | 1 | 961 | 2.32 | 0.1283 |
| black | loam | Auto | 1 | 1 | 961 | 1.23 | 0.2675 |
| black | loam | Auto | 10 | 1 | 961 | 4.27 | 0.039 |
| black | loam | Auto | 100 | 1 | 961 | 14.15 | 0.0002 |
| black | loam | Auto | 500 | 1 | 961 | 0.28 | 0.5936 |
| black | loam | Auto | 1000 | 1 | 961 | 4.34 | 0.0375 |
| black | loam | Non | 0 | 1 | 961 | 0.58 | 0.4466 |
| black | loam | Non | 1 | 1 | 961 | 0.36 | 0.5512 |
| black | loam | Non | 10 | 1 | 961 | 0.36 | 0.5484 |
| black | loam | Non | 100 | 1 | 961 | 31.4 | <.0001 |
| black | loam | Non | 500 | 1 | 961 | 47.05 | <.0001 |
| black | loam | Non | 1000 | 1 | 961 | 34.05 | <.0001 |
| brown | clay loam | Auto | 0 | 1 | 961 | 1.31 | 0.2519 |
| brown | clay loam | Auto | 1 | 1 | 961 | 2.77 | 0.0967 |
| brown | clay loam | Auto | 10 | 1 | 961 | 3.39 | 0.0657 |
| brown | clay loam | Auto | 100 | 1 | 961 | 6.45 | 0.0112 |
| brown | clay loam | Auto | 500 | 1 | 961 | 3.67 | 0.0557 |
| brown | clay loam | Auto | 1000 | 1 | 961 | 6.07 | 0.0139 |
| brown | clay loam | Non | 0 | 1 | 961 | 24.57 | <.0001 |
| brown | clay loam | Non | 1 | 1 | 961 | 19.11 | <.0001 |
| brown | clay loam | Non | 10 | 1 | 961 | 15.61 | <.0001 |
| brown | clay loam | Non | 100 | 1 | 961 | 10.09 | 0.0015 |
| brown | clay loam | Non | 500 | 1 | 961 | 12.26 | 0.0005 |
| brown | clay loam | Non | 1000 | 1 | 961 | 2.03 | 0.1542 |
| dark brown | clay loam | Auto | 0 | 1 | 961 | 3.63 | 0.057 |
| dark brown | clay loam | Auto | 1 | 1 | 961 | 0.02 | 0.9021 |
| dark brown | clay loam | Auto | 10 | 1 | 961 | 1.55 | 0.2142 |
| dark brown | clay loam | Auto | 100 | 1 | 961 | 9.88 | 0.0017 |
| dark brown | clay loam | Auto | 500 | 1 | 961 | 7.74 | 0.0055 |
| dark brown | clay loam | Auto | 1000 | 1 | 961 | 6.41 | 0.0115 |
| dark brown | clay loam | Non | 0 | 1 | 961 | 0.94 | 0.333 |
| dark brown | clay loam | Non | 1 | 1 | 961 | 0.11 | 0.7347 |
| dark brown | clay loam | Non | 10 | 1 | 961 | 1.24 | 0.2663 |
| dark brown | clay loam | non | 100 | 1 | 961 | 9.68 | 0.0019 |
| dark brown | clay loam | non | 500 | 1 | 961 | 29.27 | <.0001 |
| dark brown | clay loam | non | 1000 | 1 | 961 | 54.04 | <.0001 |
| dark brown | silt loam | auto | 0 | 1 | 961 | 3.34 | 0.0678 |
| dark brown | silt loam | auto | 1 | 1 | 961 | 6.42 | 0.0115 |
| dark brown | silt loam | auto | 10 | 1 | 961 | 11.22 | 0.0008 |
| dark brown | silt loam | auto | 100 | 1 | 961 | 0.09 | 0.7658 |
| dark brown | silt loam | auto | 500 | 1 | 961 | 1.12 | 0.2896 |
| dark brown | silt loam | auto | 1000 | 1 | 961 | 1.61 | 0.2041 |
| dark brown | silt loam | non | 0 | 1 | 961 | 9.77 | 0.0018 |
| dark brown | silt loam | non | 1 | 1 | 961 | 20.53 | <.0001 |
| dark brown | silt loam | non | 10 | 1 | 961 | 13.17 | 0.0003 |
| dark brown | silt loam | non | 100 | 1 | 961 | 0.28 | 0.5964 |
| dark brown | silt loam | non | 500 | 1 | 961 | 7.05 | 0.0081 |
| dark brown | silt loam | non | 1000 | 1 | 961 | 6.22 | 0.0128 |

Table S4. Test of effects sliced by ‘texture x oospore level x treatment x year’ to determine effect of soil zone on DSI.

| **Tests of Effect Slices for Zon*Typ*Tre*Yea(Oos) Sliced By Type*Trea*Year(Oosp)** | | | | | | | |
| --- | --- | --- | --- | --- | --- | --- | --- |
| **Type** | **Treatment** | **OosporeLevel** | **Year** | **Num DF** | **Den DF** | **F Value** | **Pr > F** |
| clay loam | Auto | 0 | 2015 | 1 | 961 | 0.61 | 0.4366 |
| clay loam | Auto | 1 | 2015 | 1 | 961 | 0.48 | 0.4889 |
| clay loam | Auto | 10 | 2015 | 1 | 961 | 1.03 | 0.3102 |
| clay loam | Auto | 100 | 2015 | 1 | 961 | 0.09 | 0.7585 |
| clay loam | Auto | 500 | 2015 | 1 | 961 | 0.04 | 0.8378 |
| clay loam | Auto | 1000 | 2015 | 1 | 961 | 0.38 | 0.5362 |
| clay loam | Non | 0 | 2015 | 1 | 961 | 15.01 | 0.0001 |
| clay loam | Non | 1 | 2015 | 1 | 961 | 15.01 | 0.0001 |
| clay loam | Non | 10 | 2015 | 1 | 961 | 9.49 | 0.0021 |
| clay loam | Non | 100 | 2015 | 1 | 961 | 5.55 | 0.0187 |
| clay loam | Non | 500 | 2015 | 1 | 961 | 9.14 | 0.0026 |
| clay loam | Non | 1000 | 2015 | 1 | 961 | 1.6 | 0.2065 |
| clay loam | Auto | 0 | 2016 | 1 | 961 | 0 | 0.9919 |
| clay loam | Auto | 1 | 2016 | 1 | 961 | 0.73 | 0.3935 |
| clay loam | Auto | 10 | 2016 | 1 | 961 | 0.17 | 0.6812 |
| clay loam | Auto | 100 | 2016 | 1 | 961 | 0.87 | 0.3516 |
| clay loam | Auto | 500 | 2016 | 1 | 961 | 1.43 | 0.2326 |
| clay loam | Auto | 1000 | 2016 | 1 | 961 | 0.79 | 0.3746 |
| clay loam | Non | 0 | 2016 | 1 | 961 | 0.07 | 0.7951 |
| clay loam | Non | 1 | 2016 | 1 | 961 | 0.04 | 0.8405 |
| clay loam | Non | 10 | 2016 | 1 | 961 | 0.04 | 0.8492 |
| clay loam | Non | 100 | 2016 | 1 | 961 | 5.15 | 0.0235 |
| clay loam | Non | 500 | 2016 | 1 | 961 | 22.97 | <.0001 |
| clay loam | Non | 1000 | 2016 | 1 | 961 | 53.63 | <.0001 |
| loam | Auto | 0 | 2015 | 1 | 961 | 1.95 | 0.1629 |
| loam | Auto | 1 | 2015 | 1 | 961 | 2.65 | 0.1039 |
| loam | Auto | 10 | 2015 | 1 | 961 | 5.67 | 0.0175 |
| loam | Auto | 100 | 2015 | 1 | 961 | 0.36 | 0.5503 |
| loam | Auto | 500 | 2015 | 1 | 961 | 0.21 | 0.6432 |
| loam | Auto | 1000 | 2015 | 1 | 961 | 0.51 | 0.4772 |
| loam | Non | 0 | 2015 | 1 | 961 | 0.08 | 0.7821 |
| loam | Non | 1 | 2015 | 1 | 961 | 0.57 | 0.4498 |
| loam | Non | 10 | 2015 | 1 | 961 | 0.11 | 0.7416 |
| loam | Non | 100 | 2015 | 1 | 961 | 0.35 | 0.5562 |
| loam | Non | 500 | 2015 | 1 | 961 | 0 | 0.9819 |
| loam | Non | 1000 | 2015 | 1 | 961 | 0 | 0.9717 |
| silt loam | Auto | 0 | 2016 | 2 | 961 | 2.13 | 0.119 |
| silt loam | Auto | 1 | 2016 | 2 | 961 | 3.65 | 0.0263 |
| silt loam | Auto | 10 | 2016 | 2 | 961 | 5.62 | 0.0038 |
| silt loam | Auto | 100 | 2016 | 2 | 961 | 1.54 | 0.216 |
| silt loam | Auto | 500 | 2016 | 2 | 961 | 4.14 | 0.0162 |
| silt loam | Auto | 1000 | 2016 | 2 | 961 | 1.86 | 0.1558 |
| silt loam | Non | 0 | 2016 | 2 | 961 | 6.7 | 0.0013 |
| silt loam | Non | 1 | 2016 | 2 | 961 | 11.89 | <.0001 |
| silt loam | Non | 10 | 2016 | 2 | 961 | 8.78 | 0.0002 |
| silt loam | Non | 100 | 2016 | 2 | 961 | 4.7 | 0.0093 |
| silt loam | Non | 500 | 2016 | 2 | 961 | 2.37 | 0.094 |
| silt loam | Non | 1000 | 2016 | 2 | 961 | 0.29 | 0.7456 |

Table S5. Test of effects sliced by ‘zone x oospore level x treatment x year’ to determine effect of soil texture on DSI.

| **Tests of Effect Slices for Zon*Typ*Tre*Yea(Oos) Sliced By Zone*Trea*Year(Oosp)** | | | | | | | |
| --- | --- | --- | --- | --- | --- | --- | --- |
| **Zone** | **Treatment** | **OosporeLevel** | **Year** | **Num DF** | **Den DF** | **F Value** | **Pr > F** |
| black | Auto | 0 | 2015 | 1 | 961 | 0.08 | 0.783 |
| black | Auto | 1 | 2015 | 1 | 961 | 0.01 | 0.9189 |
| black | Auto | 10 | 2015 | 1 | 961 | 0.6 | 0.4394 |
| black | Auto | 100 | 2015 | 1 | 961 | 0.16 | 0.6929 |
| black | Auto | 500 | 2015 | 1 | 961 | 0.04 | 0.8353 |
| black | Auto | 1000 | 2015 | 1 | 961 | 3.5 | 0.0616 |
| black | Non | 0 | 2015 | 1 | 961 | 3.44 | 0.064 |
| black | Non | 1 | 2015 | 1 | 961 | 3.93 | 0.0477 |
| black | Non | 10 | 2015 | 1 | 961 | 4.58 | 0.0325 |
| black | Non | 100 | 2015 | 1 | 961 | 4.54 | 0.0334 |
| black | Non | 500 | 2015 | 1 | 961 | 11.42 | 0.0008 |
| black | Non | 1000 | 2015 | 1 | 961 | 0.78 | 0.3788 |
| black | Auto | 0 | 2016 | 1 | 961 | 0.66 | 0.4164 |
| black | Auto | 1 | 2016 | 1 | 961 | 0.87 | 0.3516 |
| black | Auto | 10 | 2016 | 1 | 961 | 2.34 | 0.1262 |
| black | Auto | 100 | 2016 | 1 | 961 | 23.99 | <.0001 |
| black | Auto | 500 | 2016 | 1 | 961 | 0.43 | 0.5115 |
| black | Auto | 1000 | 2016 | 1 | 961 | 4 | 0.0459 |
| black | Non | 0 | 2016 | 1 | 961 | 5.29 | 0.0217 |
| black | Non | 1 | 2016 | 1 | 961 | 5.93 | 0.0151 |
| black | Non | 10 | 2016 | 1 | 961 | 11.55 | 0.0007 |
| black | Non | 100 | 2016 | 1 | 961 | 51.03 | <.0001 |
| black | Non | 500 | 2016 | 1 | 961 | 55.26 | <.0001 |
| black | Non | 1000 | 2016 | 1 | 961 | 41 | <.0001 |
| brown | Auto | 0 | 2015 | 1 | 961 | 2.44 | 0.1187 |
| brown | Auto | 1 | 2015 | 1 | 961 | 5.68 | 0.0174 |
| brown | Auto | 10 | 2015 | 1 | 961 | 5.5 | 0.0192 |
| brown | Auto | 100 | 2015 | 1 | 961 | 0.38 | 0.537 |
| brown | Auto | 500 | 2015 | 1 | 961 | 0.12 | 0.7244 |
| brown | Auto | 1000 | 2015 | 1 | 961 | 0.02 | 0.8777 |
| brown | Non | 0 | 2015 | 1 | 961 | 16.42 | <.0001 |
| brown | Non | 1 | 2015 | 1 | 961 | 12.34 | 0.0005 |
| brown | Non | 10 | 2015 | 1 | 961 | 7.63 | 0.0058 |
| brown | Non | 100 | 2015 | 1 | 961 | 8.13 | 0.0044 |
| brown | Non | 500 | 2015 | 1 | 961 | 1.55 | 0.2128 |
| brown | Non | 1000 | 2015 | 1 | 961 | 1.16 | 0.2812 |
| brown | Auto | 0 | 2016 | 1 | 961 | 3.18 | 0.0748 |
| brown | Auto | 1 | 2016 | 1 | 961 | 5.43 | 0.02 |
| brown | Auto | 10 | 2016 | 1 | 961 | 12.31 | 0.0005 |
| brown | Auto | 100 | 2016 | 1 | 961 | 19.69 | <.0001 |
| brown | Auto | 500 | 2016 | 1 | 961 | 21.61 | <.0001 |
| brown | Auto | 1000 | 2016 | 1 | 961 | 4.3 | 0.0384 |
| brown | Non | 0 | 2016 | 1 | 961 | 23.8 | <.0001 |
| brown | Non | 1 | 2016 | 1 | 961 | 25.66 | <.0001 |
| brown | Non | 10 | 2016 | 1 | 961 | 31.33 | <.0001 |
| brown | Non | 100 | 2016 | 1 | 961 | 23.98 | <.0001 |
| brown | Non | 500 | 2016 | 1 | 961 | 5.21 | 0.0227 |
| brown | Non | 1000 | 2016 | 1 | 961 | 4.24 | 0.0398 |
| dark brown | Auto | 0 | 2015 | 1 | 961 | 0.01 | 0.9319 |
| dark brown | Auto | 1 | 2015 | 1 | 961 | 1.79 | 0.1815 |
| dark brown | Auto | 10 | 2015 | 1 | 961 | 8.18 | 0.0043 |
| dark brown | Auto | 100 | 2015 | 1 | 961 | 6.9 | 0.0087 |
| dark brown | Auto | 500 | 2015 | 1 | 961 | 4.56 | 0.0331 |
| dark brown | Auto | 1000 | 2015 | 1 | 961 | 0.16 | 0.6876 |
| dark brown | Non | 0 | 2015 | 1 | 961 | 11.19 | 0.0009 |
| dark brown | Non | 1 | 2015 | 1 | 961 | 20.22 | <.0001 |
| dark brown | Non | 10 | 2015 | 1 | 961 | 17.77 | <.0001 |
| dark brown | Non | 100 | 2015 | 1 | 961 | 21.19 | <.0001 |
| dark brown | Non | 500 | 2015 | 1 | 961 | 1.43 | 0.2314 |
| dark brown | Non | 1000 | 2015 | 1 | 961 | 1.38 | 0.2404 |
| dark brown | Auto | 0 | 2016 | 1 | 961 | 0 | 0.9741 |
| dark brown | Auto | 1 | 2016 | 1 | 961 | 1.09 | 0.296 |
| dark brown | Auto | 10 | 2016 | 1 | 961 | 0.9 | 0.3419 |
| dark brown | Auto | 100 | 2016 | 1 | 961 | 32.6 | <.0001 |
| dark brown | Auto | 500 | 2016 | 1 | 961 | 13.02 | 0.0003 |
| dark brown | Auto | 1000 | 2016 | 1 | 961 | 17.56 | <.0001 |
| dark brown | Non | 0 | 2016 | 1 | 961 | 2.09 | 0.1486 |
| dark brown | Non | 1 | 2016 | 1 | 961 | 0.35 | 0.5541 |
| dark brown | Non | 10 | 2016 | 1 | 961 | 4.15 | 0.042 |
| dark brown | Non | 100 | 2016 | 1 | 961 | 54.03 | <.0001 |
| dark brown | Non | 500 | 2016 | 1 | 961 | 76.8 | <.0001 |
| dark brown | Non | 1000 | 2016 | 1 | 961 | 49.77 | <.0001 |

Table S6. Tests of effects Sliced by ‘soil zone*soil type*oospore*year’ to determine the effect of treatment (autoclaved or non-autoclaved) on DSI.

| **Tests of Effect Slices for Zon*Typ*Tre*Yea(Oos) Sliced By Zone*Type*Oospo*Year** | | | | | | | |
| --- | --- | --- | --- | --- | --- | --- | --- |
| **Zone** | **Type** | **OosporeLevel** | **Year** | **Num DF** | **Den DF** | **F Value** | **Pr > F** |
| black | loam | 0 | 2015 | 1 | 961 | 0.44 | 0.5086 |
| black | loam | 0 | 2016 | 1 | 961 | 0.01 | 0.934 |
| black | loam | 1 | 2015 | 1 | 961 | 1.47 | 0.2259 |
| black | loam | 1 | 2016 | 1 | 961 | 0.59 | 0.4437 |
| black | loam | 10 | 2015 | 1 | 961 | 2.78 | 0.0957 |
| black | loam | 10 | 2016 | 1 | 961 | 0.07 | 0.7922 |
| black | loam | 100 | 2015 | 1 | 961 | 6.6 | 0.0104 |
| black | loam | 100 | 2016 | 1 | 961 | 0.43 | 0.5117 |
| black | loam | 500 | 2015 | 1 | 961 | 4.03 | 0.045 |
| black | loam | 500 | 2016 | 1 | 961 | 23.29 | <.0001 |
| black | loam | 1000 | 2015 | 1 | 961 | 0.94 | 0.3338 |
| black | loam | 1000 | 2016 | 1 | 961 | 10.3 | 0.0014 |
| black | sandy loam | 0 | 2015 | 1 | 961 | 0.89 | 0.346 |
| black | sandy loam | 1 | 2015 | 1 | 961 | 0.45 | 0.504 |
| black | sandy loam | 10 | 2015 | 1 | 961 | 1.51 | 0.22 |
| black | sandy loam | 100 | 2015 | 1 | 961 | 0.02 | 0.8886 |
| black | sandy loam | 500 | 2015 | 1 | 961 | 2.68 | 0.1018 |
| black | sandy loam | 1000 | 2015 | 1 | 961 | 3.18 | 0.075 |
| black | silt loam | 0 | 2016 | 1 | 961 | 2.88 | 0.09 |
| black | silt loam | 1 | 2016 | 1 | 961 | 0.66 | 0.4179 |
| black | silt loam | 10 | 2016 | 1 | 961 | 3.08 | 0.0798 |
| black | silt loam | 100 | 2016 | 1 | 961 | 14.74 | 0.0001 |
| black | silt loam | 500 | 2016 | 1 | 961 | 10.46 | 0.0013 |
| black | silt loam | 1000 | 2016 | 1 | 961 | 7.28 | 0.0071 |
| brown | clay loam | 0 | 2015 | 1 | 961 | 10.05 | 0.0016 |
| brown | clay loam | 0 | 2016 | 1 | 961 | 0.72 | 0.3966 |
| brown | clay loam | 1 | 2015 | 1 | 961 | 6.01 | 0.0144 |
| brown | clay loam | 1 | 2016 | 1 | 961 | 0.13 | 0.7196 |
| brown | clay loam | 10 | 2015 | 1 | 961 | 2.46 | 0.1174 |
| brown | clay loam | 10 | 2016 | 1 | 961 | 0.39 | 0.5332 |
| brown | clay loam | 100 | 2015 | 1 | 961 | 0.09 | 0.7634 |
| brown | clay loam | 100 | 2016 | 1 | 961 | 0.21 | 0.647 |
| brown | clay loam | 500 | 2015 | 1 | 961 | 8.11 | 0.0045 |
| brown | clay loam | 500 | 2016 | 1 | 961 | 1.7 | 0.1929 |
| brown | clay loam | 1000 | 2015 | 1 | 961 | 1.49 | 0.2225 |
| brown | clay loam | 1000 | 2016 | 1 | 961 | 5.77 | 0.0164 |
| brown | loam | 0 | 2015 | 1 | 961 | 0.22 | 0.6429 |
| brown | loam | 1 | 2015 | 1 | 961 | 1.38 | 0.2403 |
| brown | loam | 10 | 2015 | 1 | 961 | 1.12 | 0.2902 |
| brown | loam | 100 | 2015 | 1 | 961 | 13.66 | 0.0002 |
| brown | loam | 500 | 2015 | 1 | 961 | 5.41 | 0.0202 |
| brown | loam | 1000 | 2015 | 1 | 961 | 0.05 | 0.8245 |
| brown | silt loam | 0 | 2016 | 1 | 961 | 6.46 | 0.0112 |
| brown | silt loam | 1 | 2016 | 1 | 961 | 7.16 | 0.0076 |
| brown | silt loam | 10 | 2016 | 1 | 961 | 2.98 | 0.0845 |
| brown | silt loam | 100 | 2016 | 1 | 961 | 0.37 | 0.5441 |
| brown | silt loam | 500 | 2016 | 1 | 961 | 1.76 | 0.1846 |
| brown | silt loam | 1000 | 2016 | 1 | 961 | 3.41 | 0.0651 |
| dark brown | clay loam | 0 | 2015 | 1 | 961 | 2.38 | 0.1236 |
| dark brown | clay loam | 0 | 2016 | 1 | 961 | 0.36 | 0.5491 |
| dark brown | clay loam | 1 | 2015 | 1 | 961 | 0.63 | 0.4263 |
| dark brown | clay loam | 1 | 2016 | 1 | 961 | 1.02 | 0.3124 |
| dark brown | clay loam | 10 | 2015 | 1 | 961 | 0.29 | 0.5933 |
| dark brown | clay loam | 10 | 2016 | 1 | 961 | 0.16 | 0.6871 |
| dark brown | clay loam | 100 | 2015 | 1 | 961 | 3.35 | 0.0677 |
| dark brown | clay loam | 100 | 2016 | 1 | 961 | 3.26 | 0.0714 |
| dark brown | clay loam | 500 | 2015 | 1 | 961 | 0.02 | 0.8958 |
| dark brown | clay loam | 500 | 2016 | 1 | 961 | 5.88 | 0.0155 |
| dark brown | clay loam | 1000 | 2015 | 1 | 961 | 0.38 | 0.5372 |
| dark brown | clay loam | 1000 | 2016 | 1 | 961 | 18.71 | <.0001 |
| dark brown | silt loam | 0 | 2015 | 1 | 961 | 3.65 | 0.0564 |
| dark brown | silt loam | 0 | 2016 | 1 | 961 | 0.71 | 0.401 |
| dark brown | silt loam | 1 | 2015 | 1 | 961 | 5.83 | 0.0159 |
| dark brown | silt loam | 1 | 2016 | 1 | 961 | 0.37 | 0.5457 |
| dark brown | silt loam | 10 | 2015 | 1 | 961 | 0.78 | 0.3777 |
| dark brown | silt loam | 10 | 2016 | 1 | 961 | 0.52 | 0.4697 |
| dark brown | silt loam | 100 | 2015 | 1 | 961 | 0.1 | 0.7528 |
| dark brown | silt loam | 100 | 2016 | 1 | 961 | 0.01 | 0.9044 |
| dark brown | silt loam | 500 | 2015 | 1 | 961 | 0.98 | 0.3229 |
| dark brown | silt loam | 500 | 2016 | 1 | 961 | 6.74 | 0.0096 |
| dark brown | silt loam | 1000 | 2015 | 1 | 961 | 0.73 | 0.3946 |
| dark brown | silt loam | 1000 | 2016 | 1 | 961 | 0.15 | 0.6998 |

TableS7. Tests of effects Sliced by ‘soil zone*soil type*treatment*year’ to determine the effect of oospore concentration on DSI.

| **Tests of Effect Slices for Zon*Typ*Tre*Yea(Oos) Sliced By Zone*Type*Treat*Year** | | | | | | | |
| --- | --- | --- | --- | --- | --- | --- | --- |
| **Zone** | **Type** | **Treatment** | **Year** | **Num DF** | **Den DF** | **F Value** | **Pr > F** |
| black | loam | Auto | 2015 | 5 | 961 | 14.06 | <.0001 |
| black | loam | Auto | 2016 | 5 | 961 | 18.43 | <.0001 |
| black | loam | Non | 2015 | 5 | 961 | 29.87 | <.0001 |
| black | loam | Non | 2016 | 5 | 961 | 3.75 | 0.0023 |
| black | sandy loam | Auto | 2015 | 5 | 961 | 21.4 | <.0001 |
| black | sandy loam | Non | 2015 | 5 | 961 | 9.97 | <.0001 |
| black | silt loam | Auto | 2016 | 5 | 961 | 22.76 | <.0001 |
| black | silt loam | Non | 2016 | 5 | 961 | 28.01 | <.0001 |
| brown | clay loam | Auto | 2015 | 5 | 961 | 12.17 | <.0001 |
| brown | clay loam | Auto | 2016 | 5 | 961 | 11.91 | <.0001 |
| brown | clay loam | Non | 2015 | 5 | 961 | 6.21 | <.0001 |
| brown | clay loam | Non | 2016 | 5 | 961 | 24.39 | <.0001 |
| brown | loam | Auto | 2015 | 5 | 961 | 23.6 | <.0001 |
| brown | loam | Non | 2015 | 5 | 961 | 28.38 | <.0001 |
| brown | silt loam | Auto | 2016 | 5 | 961 | 16.76 | <.0001 |
| brown | silt loam | Non | 2016 | 5 | 961 | 10.24 | <.0001 |
| dark brown | clay loam | Auto | 2015 | 5 | 961 | 11.49 | <.0001 |
| dark brown | clay loam | Auto | 2016 | 5 | 961 | 7.49 | <.0001 |
| dark brown | clay loam | Non | 2015 | 5 | 961 | 20.5 | <.0001 |
| dark brown | clay loam | Non | 2016 | 5 | 961 | 0.84 | 0.5206 |
| dark brown | clay loam | Auto | 2015 | 5 | 961 | 12.38 | <.0001 |
| dark brown | clay loam | Auto | 2016 | 5 | 961 | 28.71 | <.0001 |
| dark brown | clay loam | Non | 2015 | 5 | 961 | 4.32 | 0.0007 |
| dark brown | clay loam | Non | 2016 | 5 | 961 | 33.97 | <.0001 |

Table S8. Regression parameters and *R^2^* values for the regression analysis of log10 (oospores +1)/ g soil added to the soils versus the calculated concentration of log10 (oospores +1)/ g dry measured in soil using qPCR (Q) or ddPCR (DD).

| Soil zone | Soil type | Town | PCR Type | Treatment | Slope (*b_1_*) | Intercept (*b_0_*) | *R^2^* |
| --- | --- | --- | --- | --- | --- | --- | --- |
| Dark brown | Silty loam | Biggar | DD | Autoclaved | 0.65174 | -0.15750 | 0.88 |
| Dark brown | Silty loam | Biggar | DD | Non-autoclaved | 0.87826 | -0.31435 | 0.91 |
| Dark brown | Silty loam | Biggar | Q | Autoclaved | 0.79172 | -0.09674 | 0.91 |
| Dark brown | Silty loam | Biggar | Q | Non-autoclaved | 1.04262 | -0.26329 | 0.96 |
| Black | Loam | Lacombe2 | DD | Autoclaved | 0.86038 | -0.06047 | 0.97 |
| Black | Loam | Lacombe2 | DD | Non-autoclaved | 0.78232 | 0.10490 | 0.82 |
| Black | Loam | Lacombe2 | Q | Autoclaved | 0.74004 | 0.38105 | 0.96 |
| Black | Loam | Lacombe2 | Q | Non-autoclaved | 0.80373 | 0.23392 | 0.96 |
| Dark brown | Clay loam | Lethbridge2 | DD | Autoclaved | 0.61084 | 0.34374 | 0.84 |
| Dark brown | Clay loam | Lethbridge2 | DD | Non-autoclaved | 0.85372 | 0.33189 | 0.83 |
| Dark brown | Clay loam | Lethbridge2 | Q | Autoclaved | 0.66348 | 0.37933 | 0.97 |
| Dark brown | Clay loam | Lethbridge2 | Q | Non-autoclaved | 1.09375 | -0.10931 | 0.97 |
| Black | Silty loam | Melfort | DD | Autoclaved | 1.17389 | -0.40988 | 0.94 |
| Black | Silty loam | Melfort | DD | Non-autoclaved | 0.94392 | 0.28777 | 0.99 |
| Black | Silty loam | Melfort | Q | Autoclaved | 1.19137 | -0.26928 | 0.98 |
| Black | Silty loam | Melfort | Q | Non-autoclaved | 0.88580 | 0.76138 | 0.94 |
| Brown | Clay loam | Rosemary | DD | Autoclaved | 0.84716 | 0.12185 | 0.91 |
| Brown | Clay loam | Rosemary | DD | Non-autoclaved | 0.76266 | 0.62542 | 0.88 |
| Brown | Clay loam | Rosemary | Q | Autoclaved | 0.96034 | -0.09377 | 0.98 |
| Brown | Clay loam | Rosemary | Q | Non-autoclaved | 1.02538 | -0.07116 | 0.97 |
| Brown | Silty loam | Swift | DD | Autoclaved | 0.82218 | -0.05798 | 0.76 |
| Brown | Silty loam | Swift | DD | Non-autoclaved | 0.89969 | -0.27941 | 0.91 |
| Brown | Silty loam | Swift | Q | Autoclaved | 0.80673 | 0.18184 | 0.74 |
| Brown | Silty loam | Swift | Q | Non-autoclaved | 0.92228 | 0.15176 | 0.97 |

**Figure S1.** Slope (A) and intercept (B) estimates of regression equations of log_10_ (oospores + 1)/g soil (added) versus log_10 (_oospores + 1)/g soil calculated using ddPCR or qPCR analysis of DNA extracts from autoclaved or non-autoclaved soil collected from six locations in 2016. Error bars represent the upper and lower confidence levels of the treatment means. Biggar = dark brown, silt loam; Lethbridge2 = dark brown, clay loam; Lacombe2 = black, loam; Melfort = black, silt loam; Rosemary = brown, clay loam; Swift Current = brown, silt loam.


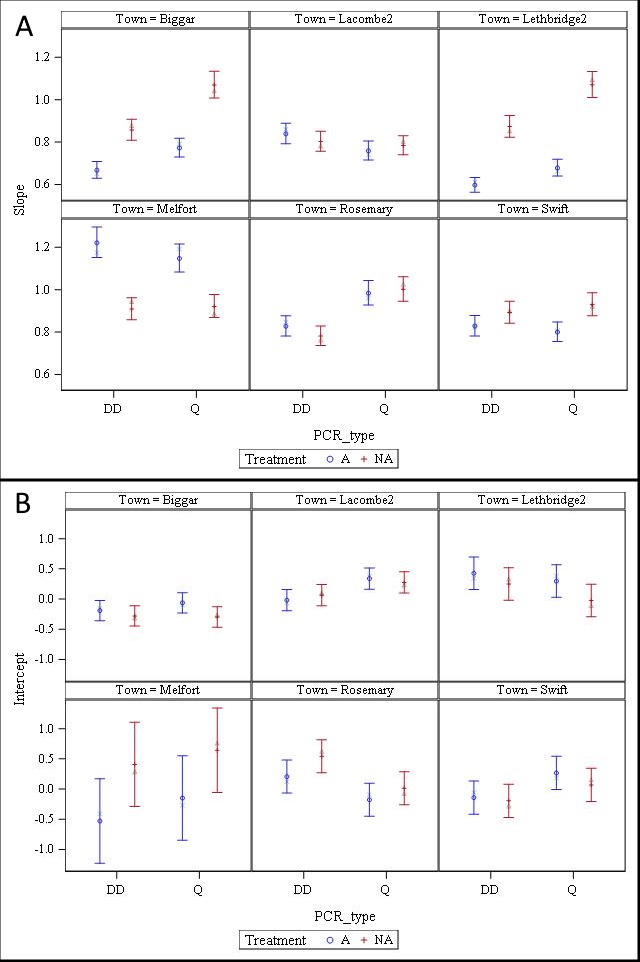

Supplement: Supplementary file 1 [file DataSheet_1.docx]
